# Circular RNA IGF1R Promotes Cardiac Repair via Activating β-Catenin Signaling by Interacting with DDX5 in Mice after Ischemic Insults

**DOI:** 10.34133/research.0451

**Published:** 2024-08-27

**Authors:** Tian-Kai Shan, Tong-Tong Yang, Peng Jing, Yu-Lin Bao, Liu-Hua Zhou, Ting Zhu, Xin-Ying Shi, Tian-Wen Wei, Si-Bo Wang, Ling-Feng Gu, Jia-Wen Chen, Ye He, Ze-Mu Wang, Qi-Ming Wang, Li-Ping Xie, Ai-Hua Gu, Yang Zhao, Yong Ji, Hao Wang, Lian-Sheng Wang

**Affiliations:** ^1^ Department of Cardiology, the First Affiliated Hospital of Nanjing Medical University, Nanjing 210029, China.; ^2^ Key Laboratory of Cardiovascular and Cerebrovascular Medicine, Key Laboratory of Targeted Intervention of Cardiovascular Disease, Collaborative Innovation Center for Cardiovascular Disease Translational Medicine, Nanjing Medical University, Nanjing, China.; ^3^ State Key Laboratory of Reproductive Medicine, School of Public Health, Nanjing Medical University, Nanjing, China.; ^4^ Department of Biostatistics, School of Public Health, China International Cooperation Center for Environment and Human Health, Nanjing Medical University, Nanjing 210029, China.

## Abstract

The potential of circular RNAs (circRNAs) as biomarkers and therapeutic targets is becoming increasingly evident, yet their roles in cardiac regeneration and myocardial renewal remain largely unexplored. Here, we investigated the function of circIGF1R and related mechanisms in cardiac regeneration. Through analysis of circRNA sequencing data from neonatal and adult cardiomyocytes, circRNAs associated with regeneration were identified. Our data showed that circIGF1R expression was high in neonatal hearts, decreased with postnatal maturation, and up-regulated after cardiac injury. The elevation was validated in patients diagnosed with acute myocardial infarction (MI) within 1 week. In human induced pluripotent stem cell-derived cardiomyocytes (hiPSC-CMs) and myocardial tissue from mice after apical resection and MI, we observed that circIGF1R overexpression enhanced cardiomyocyte proliferation, reduced apoptosis, and mitigated cardiac dysfunction and fibrosis, while circIGF1R knockdown impeded endogenous cardiac renewal. Mechanistically, we identified circIGF1R binding proteins through circRNA precipitation followed by mass spectrometry. RNA pull-down Western blot and RNA immunoprecipitation demonstrated that circIGF1R directly interacted with DDX5 and augmented its protein level by suppressing ubiquitin-dependent degradation. This subsequently triggered the β-catenin signaling pathway, leading to the transcriptional activation of cyclin D1 and c-Myc. The roles of circIGF1R and DDX5 in cardiac regeneration were further substantiated through site-directed mutagenesis and rescue experiments. In conclusion, our study highlights the pivotal role of circIGF1R in facilitating heart regeneration and repair after ischemic insults. The circIGF1R/DDX5/β-catenin axis emerges as a novel therapeutic target for enhancing myocardial repair after MI, offering promising avenues for the development of regenerative therapies.

## Introduction

Cardiovascular diseases (CVDs) represent a challenging problem in global public health. Loss of cardiomyocytes is a central issue for reduced contractile function of the heart after various ischemic and non-ischemic CVDs [1–3]. Cardiac regeneration remains a promising and attractive research hot topic in the field of CVDs. It is known that complete structural and functional recovery of myocardial tissue could be achieved in mice [4], within 7 days following cardiac insults like myocardial infarction (MI) or apical resection (AR); this regenerative capability could also be observed in neonatal porcine hearts [5]. We previously showed that ischemic adult cardiomyocytes could re-activate cell cycle activities in mice after checkpoint kinase 1 and serine/threonine-protein kinase 3 intervention [6,7]. Such insights hold the promise of unveiling novel therapeutic avenues for managing MI in adults.

Circular RNA (circRNA), a unique type of non-coding RNA, is naturally produced through backsplicing, which covalently bonds the 3′ and 5′ splice sites to create a circular structure [8–10]. This configuration provides circRNAs with greater stability and prevalence in eukaryotic cells than linear RNAs. Tissue- and cell-specific circRNAs are evolutionarily conserved, displaying dynamic expression patterns during physiological development and pathophysiological changes [11–14]. Latest studies underscore the substantial contribution of circRNAs to the pathogenesis of diverse diseases, notably cancer and CVDs [15–21]. Yet, studies on the impact of circRNAs in cardiac regeneration are limited [22–24]. Noteworthy research by Huang et al. [23] revealed that circNfix depletion, linked with super enhancers, could facilitate cardiac regenerative repair by obstructing the ubiquitin-dependent degradation of Ybx1 and enhancing miR-214 activity. Additionally, circHipk3 overexpression stimulated cardiomyocyte proliferation and alleviated cardiac dysfunction by increasing Notch1 intracellular domain acetylation [24]. Identifying novel circRNAs related to cardiac regeneration is helpful to develop innovative therapeutic agents to promote heart repair.

In this study, circRNA profiling, quantitative polymerase chain reaction (qPCR), and in situ hybridization were carried out to identify novel circRNAs related to cardiac repair. The circRNA’s role in cardiac regeneration after ischemic injury was evaluated through gain- and loss-of-function experiments conducted. Furthermore, circRNA–protein interactions were identified using RNA pull-down and RNA immunoprecipitation (RIP), along with coimmunoprecipitation (Co-IP) assays for protein–protein interaction analysis. Through the application of the above techniques, we identified a marked elevation of circIGF1R in neonatal mouse hearts in contrast to adult counterparts, with further increases following cardiac injuries such as AR or MI. In patients with acute myocardial infarction (AMI) within 1 week, plasma sample analyses showed a widespread up-regulation of circIGF1R. Functional experiments demonstrated that overexpressing circIGF1R promoted CM proliferation and inhibited CM apoptosis. Further exploration of molecular mechanisms revealed that circIGF1R could interact with DDX5 and activate the β-catenin signaling pathway.

## Results

### circIGF1R is involved in endogenous cardiac regeneration

**Fig. 1.**
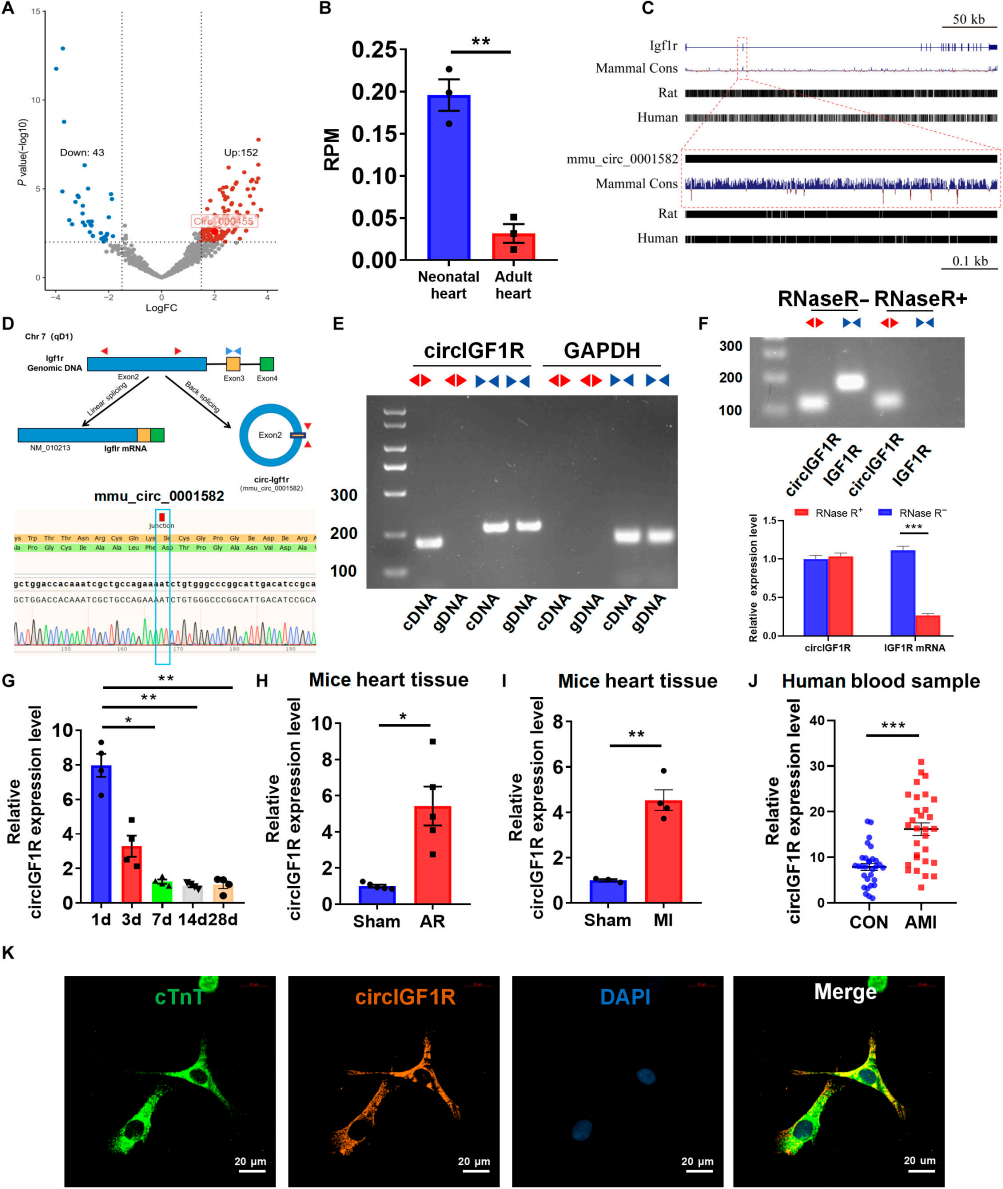
CircIGF1R is highly expressed in neonatal hearts and up-regulated in response to AR and MI model. (A) Volcano plot of the differentially expressed circRNAs between the neonatal and the adult rat hearts. (B) The expression level of rnoCirc_000455 is shown in RPM (reads per million mapped reads). (C) Species conservation analysis of the circIGF1R sequence using the UCSC Genome Browser and BLAST Browser. (D and E) The circular structure of circIGF1R was confirmed by amplification from gDNA and cDNA with both convergent and divergent primers using agarose gel electrophoresis, followed by Sanger sequencing. (F) CircIGF1R and IGF1R mRNA expression levels in CMs determined by PCR after RNase R treatment. (G) Relative expression of circIGF1R determined by qRT-PCR in hearts of mice at different ages, *n* = 4 in each group. (H and I) Relative expression of circIGF1R determined by qRT-PCR in hearts of mice after AR and MI, *n* = 4 in each group. (J) Relative expression of circIGF1R determined by qRT-PCR in the plasma of patients with or without AMI within 7 days, *n* = 30 in each group. (K) RNA-FISH assays of circIGF1R distribution in P1 CMs. Scale bars, 20 μm. Data are presented as mean ± SEM. *P ≤ 0.05, **P ≤ 0.01, and ***P ≤ 0.001.

It is widely recognized that neonatal mice hearts exhibit the remarkable capacity for myocardial regeneration shortly after birth, a capacity conspicuously absent in adult [4,25]. To explore the function of circRNAs in this regenerative capacity, we analyzed previously published transcriptomic datasets to contrast circRNA expression profiles between neonatal and adult cardiomyocytes [26]. Our analysis indicated a marked up-regulation of rnoCirc_000455, also known as circIGF1R (circular insulin like growth factor 1 receptor), in neonatal rat hearts (postnatal day 1 [P1]) compared to adults (Fig. 1A and B). CircIGF1R is originated from the second exon of the *Igf1r* (Fig. 1D). In circBase, the circRNA IDs for circIGF1R in human and mouse genomes are hsa_circ_0005035 and mmu_circ_0001582, respectively. Comparisons of circIGF1R sequence across species showed a high degree of conservation, and the homology in mice, rats, and humans is over 94% (Fig. 1C and Fig. S1A and B). To confirm the circular nature of circIGF1R, we utilized divergent primers for amplification from cDNA, and convergent primers for amplification of its linear mRNA counterpart from both cDNA and genomic DNA in neonatal mouse cardiomyocytes (NMCMs). PCR analysis confirmed the exclusive amplification of circIGF1R by divergent primers in cDNA, not in genomic DNA, thereby confirming its circular structure (Fig. 1E). This finding was further substantiated by Sanger sequencing, which aligned with the sequence reported in circBase (Fig. 1D). The stability of circIGF1R was further determined by RNase R treatment, and the results showed that circIGF1R was resistant to RNase R digestion, instead of linear transcript (Fig. 1F). Furthermore, we analyzed the expression of circIGF1R across multiple stages of cardiac development. In accordance with our RNA sequencing findings, expression of circIGF1R was markedly higher in neonatal (P1) hearts compared to those observed at P7 or in adulthood (Fig. 1G). Additionally, circIGF1R expression was re-induced following regenerative injuries such as AR or MI (Fig. 1H and I). Subsequently, the comparative analysis of circIGF1R expression in plasma samples of AMI patients and healthy controls showed an obvious elevation of circIGF1R in patients with AMI (Fig. 1J). Furthermore, the results of quantitative real-time polymerase chain reaction (qRT-PCR) and fluorescence in situ hybridization (FISH) assays ascertained that circIGF1R was primarily enriched within the cytoplasm of NMCMs (Fig. 1K and Fig. S2A). Moreover, in adult hearts at 72 h following MI, the considerable up-regulation of circIGF1R in the ischemic zone and border zone indicated its potential role in the myocardial repair process (Fig. S2B).

### circIGF1R mediates CM proliferation in NMCMs and hiPSC-CMs

**Fig. 2.**
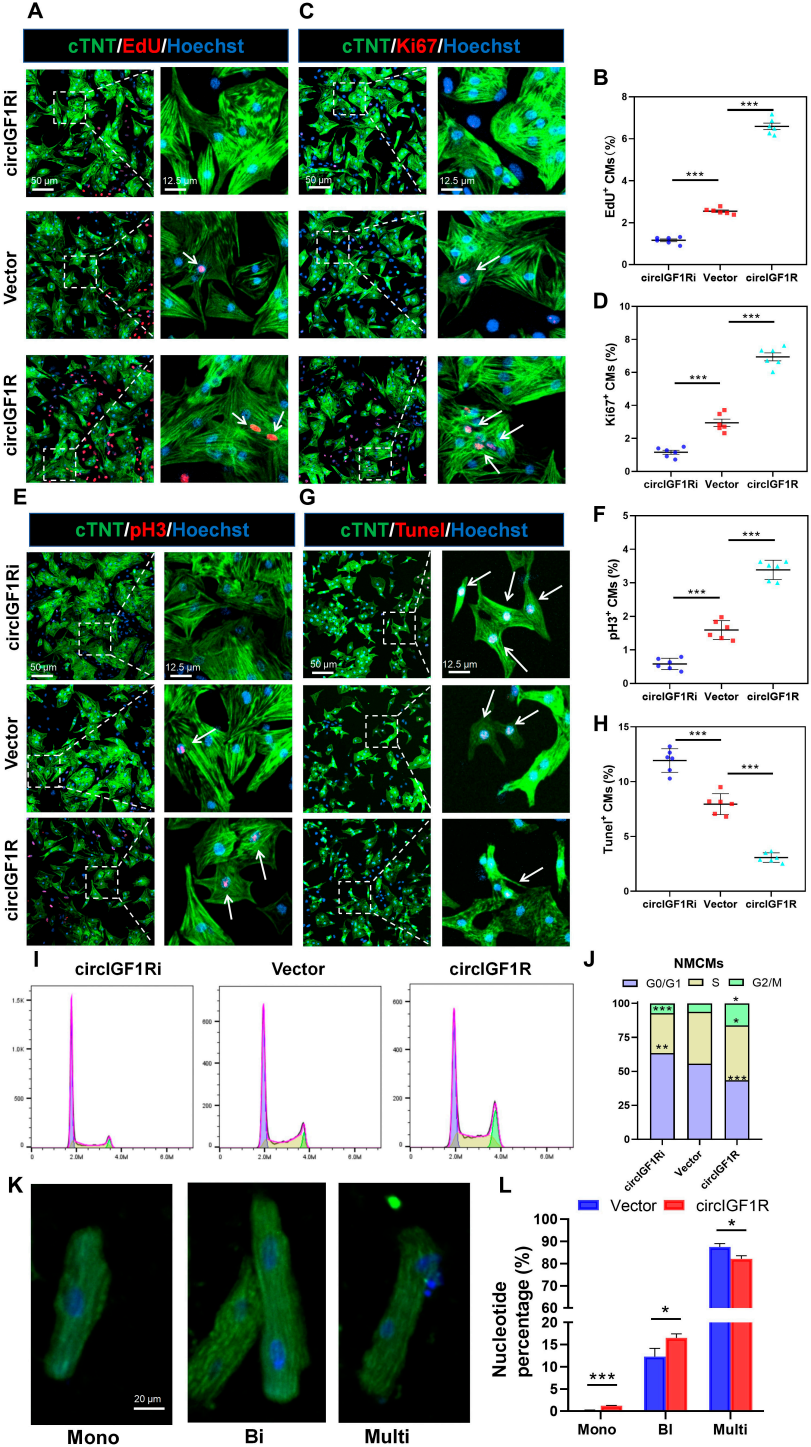
CircIGF1R mediates mice CM proliferation and apoptosis in vitro. (A to F) Representative pictures and quantification analysis of CM proliferation quantified by immunofluorescence for DNA synthesis (EdU), cell-cycle activity (Ki67), and mitosis (pH3) in P1 NMCMs transfected with Ad5: cTNT-sicircIGF1R, Ad5: cTNT-CON, and Ad5: cTNT-circIGF1R, *n* = 6 in each group. Scale bars, 50 and 12.5 μm. (G and H) Representative pictures and quantification analysis of CM apoptosis quantified by TUNEL staining in P1 NMCMs transfected with Ad5: cTNT-sicircIGF1R, Ad5: cTNT-CON, and Ad5: cTNT-circIGF1R after oxygen glucose deprivation (OGD), *n* = 6 in each group. Scale bars, 50 and 12.5 μm. (I and J) Cell flow cytometry was performed to detect the cell cycle of P1 NMCMs transfected with Ad5: cTNT-sicircIGF1R, Ad5: cTNT-CON, and Ad5: cTNT-circIGF1R, *n* = 3 in each group. (K and L) Representative pictures and quantification analysis of nucleation (mono-, bi-, and multi-) of primary P56 adult mouse cardiomyocytes (AMCMs) transfected with Ad5: cTNT-CON and Ad5: cTNT-circIGF1R, *n* = 3 in each group. Scale bar, 20 μm. Positive staining cardiomyocytes were indicated by arrows. Picture in each rectangular box was enlarged in the neighboring right panel. Data are presented as mean ± SEM. *P ≤ 0.05, **P ≤ 0.01, and ***P ≤ 0.001.

To unveil the impact of circIGF1R on modulating cardiomyocyte proliferation in vitro, we engineered adenoviruses Ad5: cTNT-sicircIGF1R and Ad5: cTNT-circIGF1R for targeted knockdown and overexpression of circIGF1R, respectively. We initially isolated cardiomyocytes from neonatal mouse hearts transfected with the aforementioned adenoviruses, observing a substantial reduction or increase in circIGF1R expression (Fig. S3A and B). Our experiments in NMCMs indicated that circIGF1R overexpression markedly increased the proportion of cardiomyocytes undergoing proliferation, as evidenced by elevated expression of EdU, Ki67, and pH3, markers of DNA synthesis, cell cycle progression, and mitosis, respectively. Additionally, this overexpression corresponded with a decreased proportion of apoptotic cardiomyocytes. In contrast, circIGF1R suppression resulted in a decrease in CM proliferation and an increasing number of apoptotic cells (Fig. 2A to H). Flow cytometry analysis unveiled that circIGF1R knockdown markedly reduced the proportion of myocardial cells in the S and G2/M phases, while overexpression had a reverse effect, enhancing the proportion of cells in these critical phases (Fig. 2I and J). We also isolated cardiomyocytes from adult mouse hearts transfected with Ad5: cTNT-CON and Ad5: cTNT-circIGF1R, noting that the proportion of mononuclear CMs was remarkably boosted by circIGF1R overexpression when compared to the control, as shown in Fig. 2K and L.

**Fig. 3.**
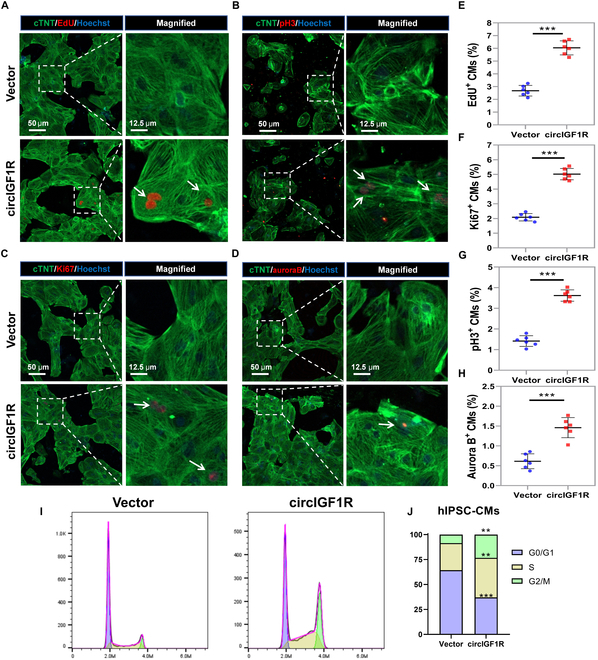
Overexpression of circIGF1R promotes human IPSC-CM proliferation. (A to H) Representative pictures and quantification analysis of CM proliferation quantified by immunofluorescence for DNA synthesis (EdU), cell-cycle activity (Ki67), mitosis (pH3), and cytokinesis (Aurora B) in human IPSC-CMs transfected with Ad5: cTNT-CON and Ad5: cTNT-circIGF1R, *n* = 6 in each group. Scale bars, 50 and 12.5 μm. (I and J) Cell flow cytometry was performed to detect the cell cycle of human IPSC-CMs transfected with Ad5: cTNT-CON and Ad5: cTNT-circIGF1R, *n* = 3 in each group. Positive staining cardiomyocytes were indicated by arrows. Picture in each rectangular box was enlarged in the neighboring right panel. Data are presented as mean ± SEM. *P ≤ 0.05, **P ≤ 0.01, and ***P ≤ 0.001.

Considering the substantial homology of circIGF1R between human and mouse, we extended our investigation to its regulatory role in human cardiomyocyte proliferation. Similar to our observations in mouse cardiomyocytes, circIGF1R overexpression in hiPSC-CMs resulted in marked cell proliferation enhancement. Immunofluorescent staining showed an increased ratio of proliferative hiPSC-CMs expressing EdU, Ki67, pH3, and Aurora B, as illustrated in Fig. 3A to H. Flow cytometry further substantiated that targeted circIGF1R effectively elevated the percentage of S and G2/M phase cardiomyocytes (Fig. 3I and J). These results collectively highlight the crucial role of circIGF1R in facilitating proliferation and reducing apoptosis of cardiomyocytes.

### circIGF1R overexpression promotes CM proliferation in neonatal mice

The effects of circIGF1R on cardiomyocyte proliferation and size in neonatal mice were studied by administering AAV9: cTNT-circIGF1R or AAV9: cTNT-CON through intraperitoneal injection to P1 mice, as outlined in Fig. S4A. Fourteen and 22 days subsequent to administration, the hearts were excised to assess circIGF1R expression and its cardiac implications. Survival analysis up to 22 days post-injection showed no significant difference between the groups (Fig. S4B). The echocardiography results indicated similar cardiac function between 2 groups (Fig. S4C and D). Further, immunofluorescence staining at P14 disclosed a substantial augmentation in the proportion of mitotic (pH3^+^) CMs in the AAV9: cTNT-circIGF1R cohort (Fig. S4E). Nevertheless, the CM size, heart weight-to-body weight ratio at P14, and cardiac fibrosis at P22 did not differ significantly between groups injected with AAV9: cTNT-circIGF1R and AAV9: cTNT-CON (Fig. S4F to H).

### Knockdown of circIGF1R prevents cardiac regeneration following AR

**Fig. 4.**
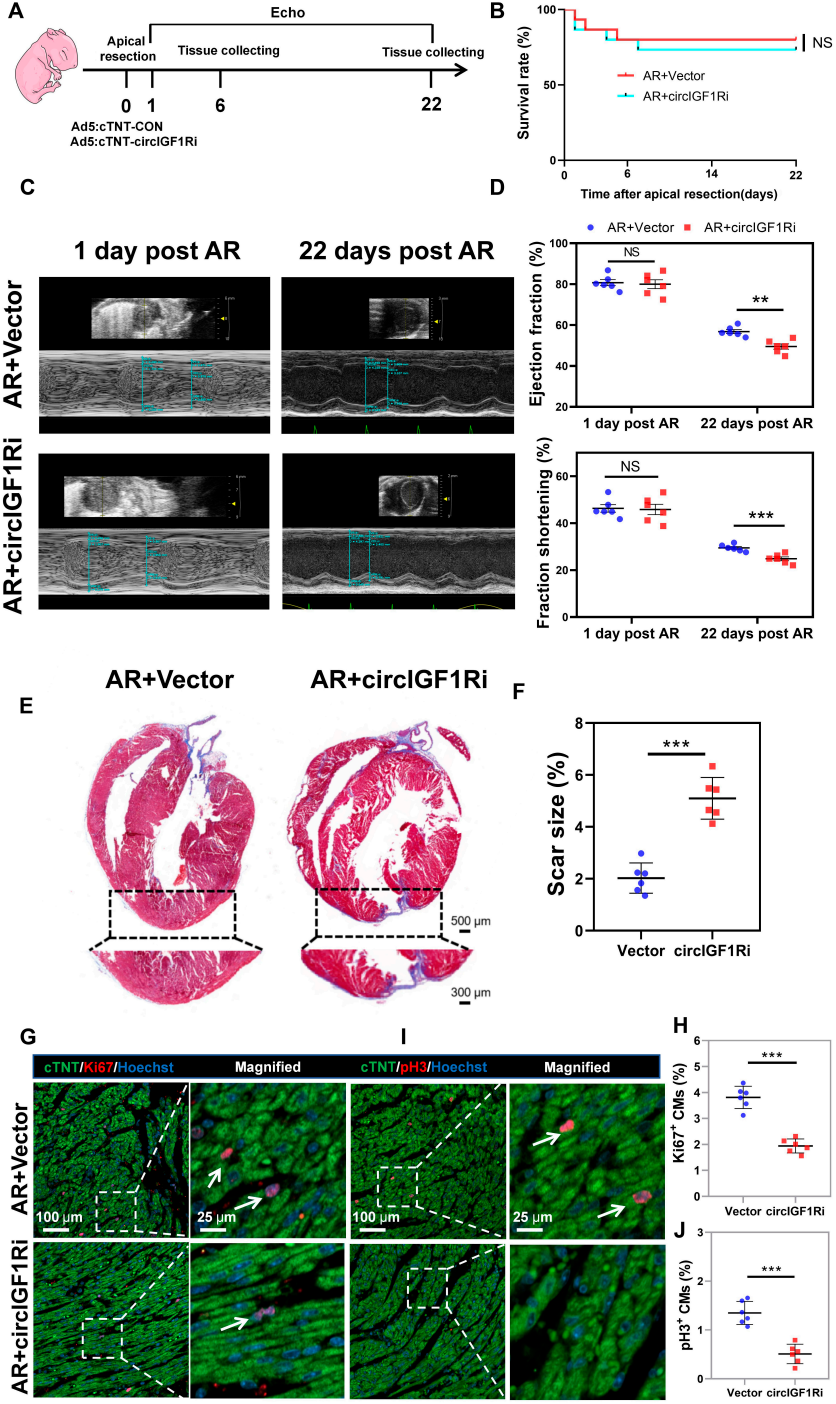
Knockdown of circIGF1R prevents neonatal mice cardiomyocyte proliferation following AR. (A) Experimental pattern: Ad5: cTNT-CON or Ad5:cTNT-sicircIGF1R was injected into myocardium following AR in P1 mice. Hearts were harvested at 6 and 22 days post-resection (dpr) to evaluate cardiomyocyte proliferation and scar area, respectively. Echocardiography was performed at 1 and 22 dpr to detect cardiac function. (B) Overall survival rate in mice treated with Ad5:cTNT-CON or Ad5:cTNT-sicircIGF1R, *n* = 20 in each group. (C and D) Cardiac function of ejection fraction and fractional shortening among the Ad5: cTNT-CON and Ad5: cTNT-sicircIGF1R treated mice at 1 and 22 day post-operation were detected by echocardiography, *n* = 6 in each group. (E and F) Masson’s trichrome staining was used to determine scar formation between the Ad5: cTNT-CON and Ad5: cTNT-sicircIGF1R treated mice at 22 dpr, *n* = 6 in each group. Scale bars, 500 μm and 300 μm. (G to J) Representative pictures and quantification analysis of CM proliferation quantified by cell-cycle activity (Ki67) and mitosis (pH3) in infarct border zone in Ad5: cTNT-CON and Ad5: cTNT-sicircIGF1R groups after AR, *n* = 6 in each group. Scale bars, 100 and 25 μm. Positive staining cardiomyocytes were indicated by arrows. Picture in each rectangular box was enlarged in the neighboring right panel. Data are presented as mean ± SEM. NS, no significance. *P ≤ 0.05, **P ≤ 0.01, and ***P ≤ 0.001.

We further explore the influence of circIGF1R on neonatal cardiac regeneration. The adenovirus-mediated silencing of circIGF1R (Ad5: cTNT-sicircIGF1R) and a control virus (Ad5: cTNT-CON) were injected directly into the cardiac apex post-AR in P1 mice, as outlined in Fig. 4A. Survival analysis up to 22 days post-AR showed no significant difference between the groups (Fig. 4B), suggesting that circIGF1R knockdown does not impact short-term survival. QRT-PCR assays confirmed effective silencing of circIGF1R expression in the myocardium of mice treated with Ad5: cTNT-sicircIGF1R compared to the control group (Fig. S5A). Echocardiographic evaluations demonstrated that circIGF1R knockdown detrimentally affected cardiac functional recovery post-AR (Fig. 4C and D and Fig. S5B and C). Masson’s trichrome staining showed a marked augmentation in cardiac fibrosis in circIGF1R-deficient mice compared to controls (Fig. 4E and F). Moreover, immunofluorescence staining displayed a notable reduction in cardiomyocyte proliferation at the resection border zone, indicated by diminished numbers of Ki67^+^ and pH3^+^ cardiomyocytes (Fig. 4G to J). In summary, our findings unequivocally demonstrate that circIGF1R knockdown inhibits cardiomyocyte proliferation, thereby impairing regenerative capacity of the neonatal mouse hearts.

### circIGF1R overexpression promotes cardiac regenerative repair in adult mice following MI

**Fig. 5.**
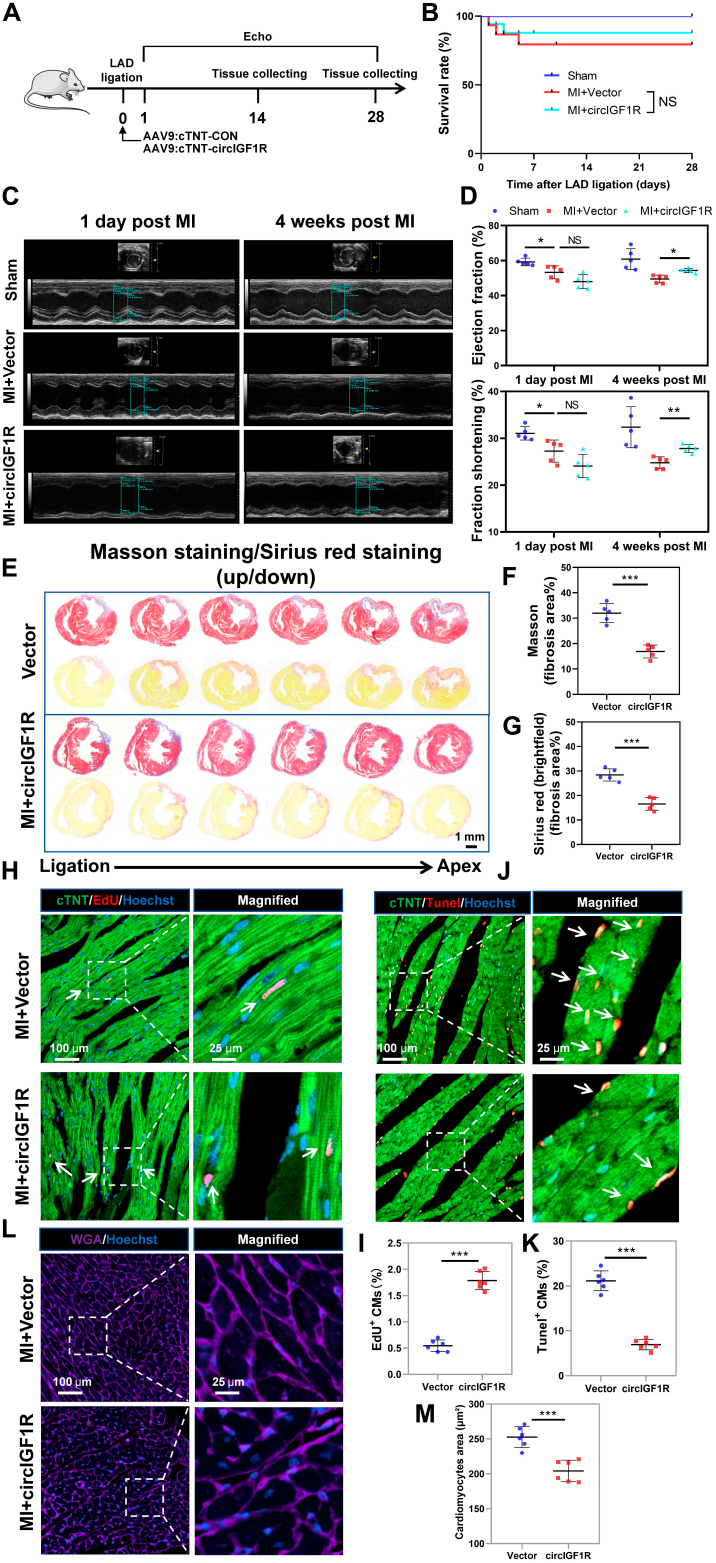
CircIGF1R promotes cardiac regeneration and repair in adult mice after MI. (A) Experimental pattern: AAV9: cTNT-CON or AAV9: cTNT-circIGF1R was injected into myocardium following MI in P56 mice. Hearts were harvested at 14 and 28 dpi to evaluate cardiomyocyte proliferation, apoptosis, and scar area, respectively. Echocardiography was performed at 1 and 28 dpi to detect cardiac function. (B) Overall survival rate in mice treated with AAV9: cTNT-CON or AAV9: cTNT-circIGF1R, *n* = 20 in each group. (C and D) Cardiac function of ejection fraction and fractional shortening among the sham-, AAV9: cTNT-CON-, or AAV9: cTNT-circIGF1R-treated mice at 1 and 28 day post-operation were detected by echocardiography, *n* = 5 in each group. (E to G) Masson’s trichrome staining and Sirius red staining were used to determine scar formation between the AAV9: cTNT-CON- or AAV9: cTNT-circIGF1R-treated mice at 28 dpi, *n* = 5 in each group. Scale bar, 1 mm. (H and I) Representative pictures and quantification analysis of CM proliferation quantified by DNA synthesis (EdU) in the infarct border zone in AAV9: cTNT-CON or AAV9: cTNT-circIGF1R groups after MI, *n* = 6 in each group. (J and K) Representative pictures and quantification analysis of CM apoptosis quantified by TUNEL staining in the infarct border zone in AAV9: cTNT-CON or AAV9: cTNT-circIGF1R groups after MI, *n* = 6 in each group. (L and M) Representative pictures and quantification analysis of CM size quantified by WGA immunofluorescence in the infarct border zone in AAV9: cTNT-CON or AAV9: cTNT-circIGF1R groups after MI, *n* = 6 in each group. Scale bars, 100 and 25 μm. Positive staining cardiomyocytes were indicated by arrows. Picture in each rectangular box was enlarged in the neighboring right panel. Data are presented as mean ± SEM. NS, no significance. *P ≤ 0.05, **P ≤ 0.01, and ***P ≤ 0.001.

Additionally, we explored the therapeutic potential of circIGF1R overexpression in adult mice post-MI. CircIGF1R overexpression was achieved via peri-infarct injection of AAV9: cTNT-circIGF1R, with AAV9: cTNT-CON serving as the control, into the myocardium post-MI (detail in Fig. 5A). QRT-PCR assays confirmed effective overexpression of circIGF1R in the myocardium of mice treated with AAV9: cTNT-circIGF1R compared to the control group (Fig. S6A). Survival rates post-MI were similar across the 3 groups at 28 days post-MI (dpi), as illustrated in Fig. 5B. Cardiac function was assessed non-invasively through echocardiography, with mice treated with AAV9: cTNT-circIGF1R showing significant improvements in cardiac function (Fig. 5C and D and Fig. S6B and C). Further histological analyses, including Masson’s trichrome and Sirius red staining, suggested a protective effect of circIGF1R overexpression against MI-induced cardiac fibrosis (Fig. 5E to G). Corroborating our in vitro findings, circIGF1R overexpression significantly augmented the population of proliferative cardiomyocytes, marked by EdU, Ki67, and pH3 positivity, and mitigated apoptosis within the infarct border zone at 14 dpi, as compared to controls (Fig. 5H to K and Fig. S6D to G). Moreover, wheat germ agglutinin (WGA) staining revealed a reduction in the cardiomyocyte area at the border zone in circIGF1R overexpression mice (Fig. 5L and M).

### CircIGF1R physically interacts with DDX5 protein and blocks its degradation via the ubiquitin–proteasomal pathway

**Fig. 6.**
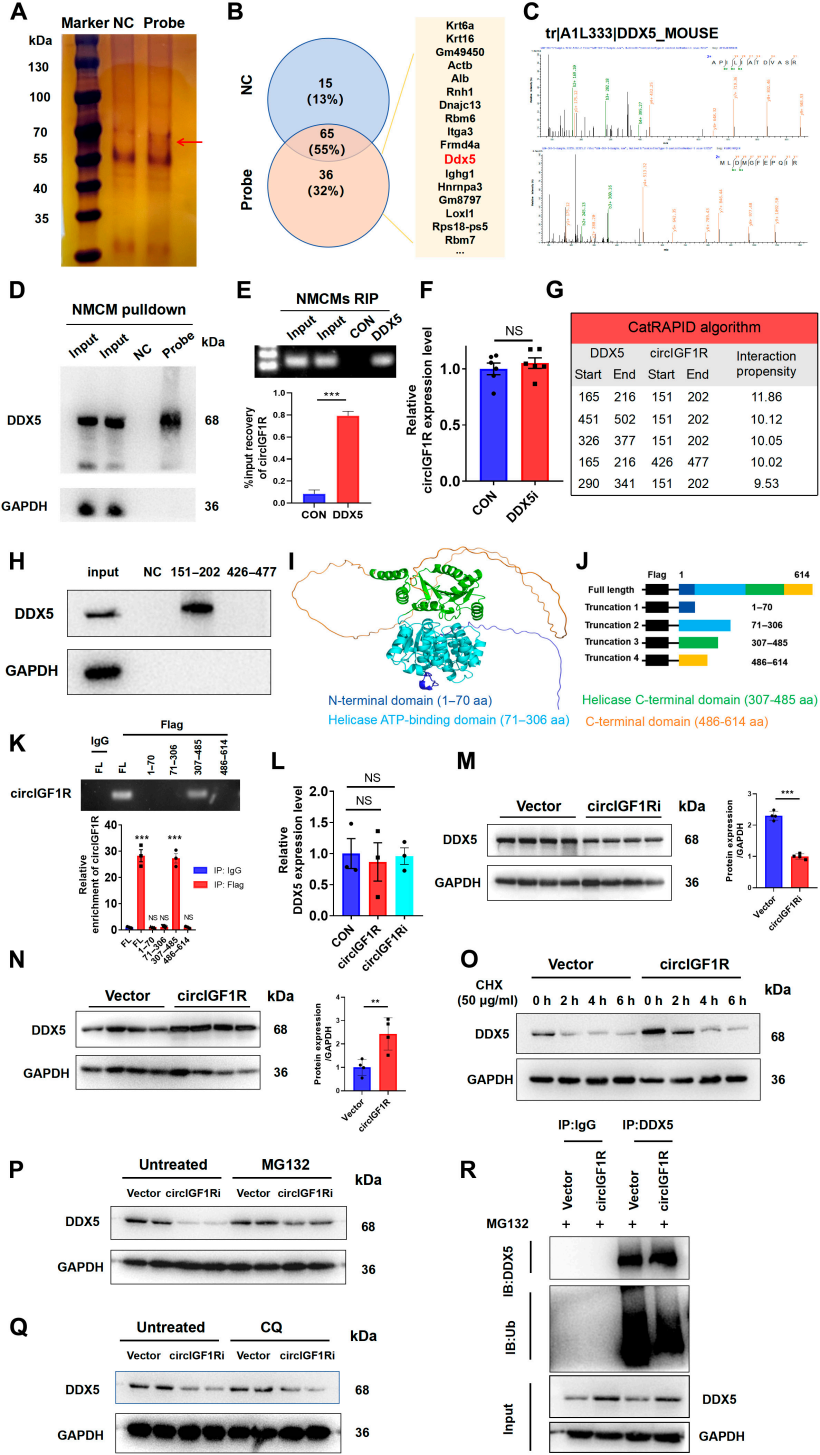
CircIGF1R physically interacts with DDX5 protein and blocks its degradation via the ubiquitin–proteasomal pathway. (A) Proteins immunoprecipitated by the specific probe targeting the circIGF1R back-splice site were detected by silver staining. (B) Venn diagram of proteins interacting with circIGF1R, as identified by RNA pull-down assay and LC-MS/MS. (C) DDX5-specific peptide sequence identified using LC-MS/MS. (D) Western blot analysis following the RNA pull-down assay revealed the interaction between circIGF1R and DDX5, *n* = 3 in each group. (E) RIP assays confirmed that the DDX5 protein interacts with circIGF1R in CMs, *n* = 3 in each group. (F) Relative expression of circIGF1R determined by qRT-PCR in NMCMs transfected with Ad5: cTNT-CON and Ad5: cTNT-shDDX5.* n* = 6 in each group. (G) Prediction of circIGF1R-DDX5 interaction using the catRAPID algorithm. (H) Western blot analysis following the RNA pull-down assay verified the binding sequence of circIGF1R by using 2 biotinylated probes (151 to 202 and 426 to 477). (I) Visualized spatial structure of DDX5 protein using AlphaFold with functional protein domains. (J) Diagrams of full-length (FL) DDX5 proteins and truncations with domain depletion. (K) Binding domain of DDX5 for circIGF1R identified by an RIP assay. (L) qRT-PCR assays determining the relative mRNA expression levels of DDX5 after circIGF1R overexpression or knockdown.* n* = 3 in each group. (M and N) Western blot analysis determining the relative protein expression levels of DDX5 after circIGF1R overexpression or knockdown. * n* = 4 in each group. (O) Western blot analysis determining the expression of DDX5 in circIGF1R-overexpressed NMCMs treated with CHX (50 μg/ml). *n* = 3 in each group. (P and Q) Western blot analysis determining the expression of DDX5 in circIGF1R-silenced NMCMs treated with MG132 or CQ.* n* = 3 in each group. (R) Western blot analysis following Co-IP assay of the ubiquitination levels of DDX5 after circIGF1R overexpression. Data are presented as mean ± SEM. NS, no significance. *P ≤ 0.05, **P ≤ 0.01, and ***P ≤ 0.001.

We further investigated the mechanism by which circIGF1R modulates cardiomyocyte proliferation and apoptosis. Given the known protein-binding capacity of several circRNAs, we utilized RNA pull-down experiments to detect potential protein interactors with a biotin-labeled probe that targets the circIGF1R backsplice sequence. Silver staining revealed a distinct band of approximately 70 kDa in the probe-treated group rather than the control, as illustrated in Fig. 6A. Mass spectrometry (MS) analysis of the precipitated proteins identified a substantial up-regulation of 36 proteins in the probe group (Table S2). Notably, both MS and subsequent Western blotting post-RNA pull-down corroborated the identification of DEAD-box helicase 5 (DDX5) as a binding protein with circIGF1R (Fig. 6B to D). Additionally, anti-DDX5 antibody-mediated RIP assays have successfully validated the interplay between circIGF1R and DDX5 (Fig. 6E). DDX5 was previously recognized for its involvement in circRNA biogenesis [27]. To assess whether circIGF1R was modulated by DDX5, we conducted shRNAs to knock down DDX5 and then assessed the expression of circIGF1R. The results revealed no discernible down-regulation of circIGF1R following DDX5 knockdown (Fig. 6F). To further verify the precise binding sites between circIGF1R and DDX5, we utilized the catRAPID algorithm (http://service.tartaglialab.com) to predict the interacting regions [28]. According to the interaction scores, the regions spanning 151 to 202 nt and 426 to 477 nt of circIGF1R were identified as necessary for interaction with DDX5 (Fig. 6G and Table S4). RNA pull-down assays demonstrated that a biotinylated probe targeting the 151 to 202 nt region successfully pulled down DDX5 protein, while a probe targeting the 426 to 477 nt region did not (Fig. 6H). This result indicates that the 151 to 202 nt region of circIGF1R is crucial for its interaction with DDX5. To pinpoint the circIGF1R binding region on DDX5, we divided DDX5 into 4 domains based on AlphaFold3 (https://alphafold.com) predictions and previous studies [29]: the N-terminal domain (1 to 70 amino acids [aa]), the helicase adenosine triphosphate (ATP)-binding domain (71 to 306 aa), the helicase C-terminal domain (307 to 485 aa), and the C-terminal domain (486 to 614 aa) (Fig. 6I). We next constructed Flag-tagged full-length DDX5 and its truncated mutants according to these functional domains (Fig. 6J). An anti-Flag RIP assay revealed that only the truncated mutant comprising the 307 to 485 aa region was able to enrich circIGF1R, whereas the 1 to 70 aa, 71 to 306 aa, or 486 to 614 aa truncated mutants did not (Fig. 6K). These results indicate that circIGF1R binds to the helicase C-terminal domain (307 to 485 aa) of DDX5 via its 151 to 202 nt fragment. The above results suggested that DDX5 might function as a scaffolding protein of circIGF1R, facilitating the regulatory effects of circIGF1R on its downstream targets.

Following the validation of the molecular interaction sites, we examined the specific regulatory mechanism of circIGF1R on DDX5 expression. The results demonstrated that DDX5 mRNA levels remained unchanged by either overexpression or knockdown of circIGF1R (Fig. 6L). However, Western blot analysis revealed that increased circIGF1R expression led to a marked up-regulation of DDX5 protein, whereas knockdown of circIGF1R resulted in reduced DDX5 expression, suggesting that circIGF1R may regulate DDX5 at the post-transcriptional level (Fig. 6M and N). To further elucidate the mechanism by which circIGF1R regulates DDX5 expression, we employed the protein synthesis inhibitor cycloheximide (CHX) to observe the effect of circIGF1R on DDX5 degradation. Western blot analysis revealed that DDX5 exhibited an extended half-life in NMCMs overexpressing circIGF1R, suggesting that circIGF1R enhanced the stability of the DDX5 protein (Fig. 6O). Additionally, the proteasome inhibitor MG132 rescued the reduction of DDX5 caused by circIGF1R inhibition in NMCMs, rather than the lysosome inhibitor Chloroquine (CQ), indicating that circIGF1R increases DDX5 protein level by reducing its proteasomal degradation (Fig. 6P to Q). Ubiquitination is a prevalent form of protein modification, with most functional proteins in cells undergoing degradation via the ubiquitin–proteasomal pathway [30]. Through ubiquitination analysis, we examined whether circIGF1R-mediated inhibition of DDX5 degradation was ubiquitination-dependent. Specifically, overexpression of circIGF1R obviously reduced the ubiquitination signal of endogenous DDX5, compared to the control group (Fig. 6R). Together, the above evidence suggests that circIGF1R directly binds to the DDX5 protein and enhances its stability in cardiomyocytes through the ubiquitin–proteasome system.

The existing literature has reported the contribution of DDX5 in cellular proliferation, differentiation, migration, and apoptosis across various tumor types. Nevertheless, DDX5’s contribution to modulating proliferation and apoptosis of CM remains to be extensively investigated. Initially, we conducted a comparative analysis of DDX5 expression in plasma samples of patients with AMI and healthy individuals utilizing qRT-PCR. Our findings uncovered a notable elevation in DDX5 mRNA levels in AMI patients in comparison to the control group (Fig. S7A). Subsequently, we examined DDX5 in mouse hearts at different stages by Western blot. Figure S7B and C shows that DDX5 expression was obviously elevated during cardiac regeneration phases, with minimal expression detected outside these periods. Furthermore, DDX5 expression markedly increased following regenerative injuries such as AR (Fig. S7D and E). These findings preliminarily hint a potential link between DDX5 and cardiac regeneration. To determine whether DDX5 directly influences CM proliferation, we overexpressed DDX5 in NMCMs, and the results demonstrated that DDX5 overexpression substantially promotes myocardial regeneration and reduces apoptosis (Fig. S7F to I).

### CircIGF1R up-regulates β-catenin signaling pathway and promotes transcriptional activation of c-Myc/cyclin D1

**Fig. 7.**
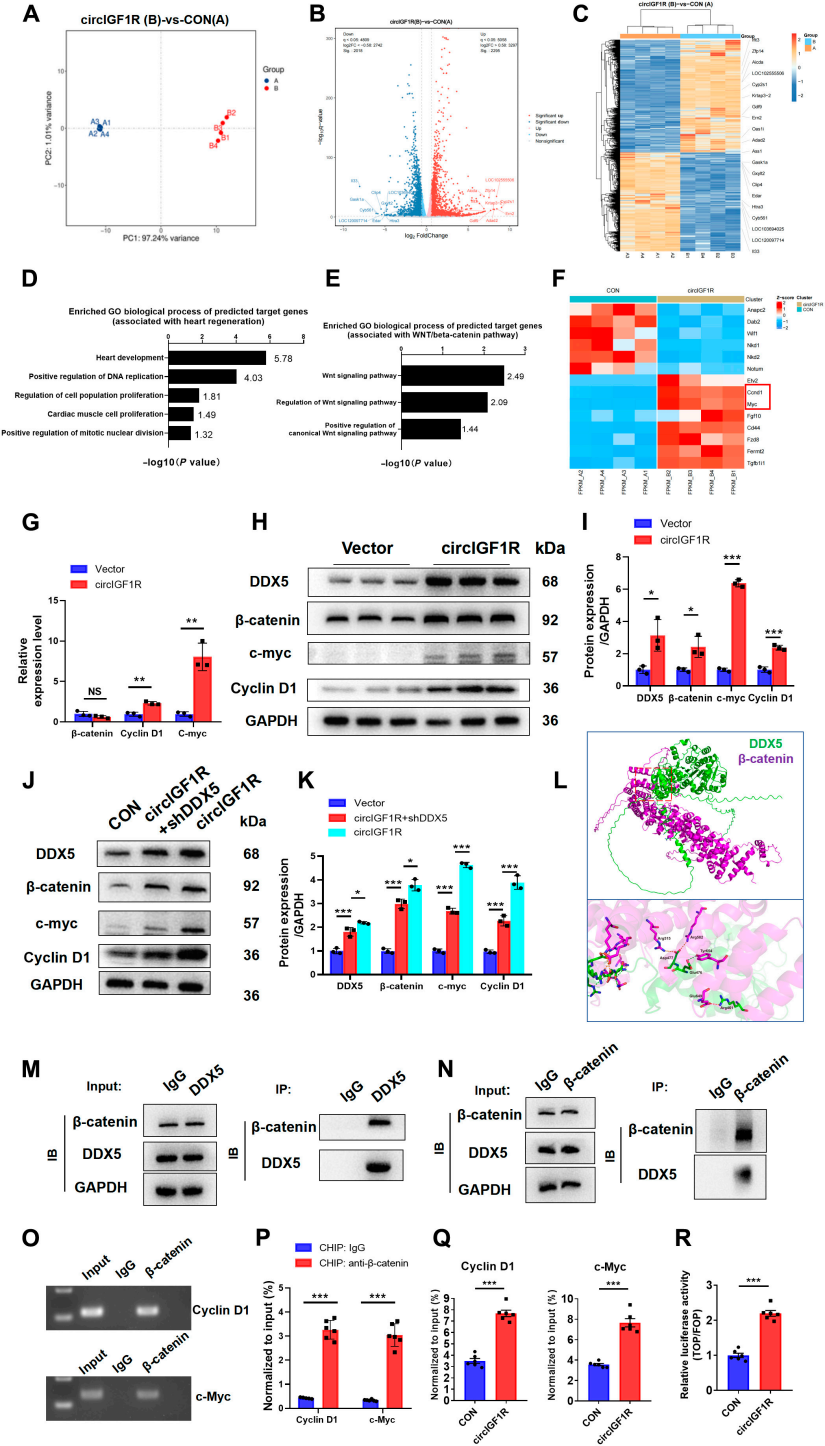
CircIGF1R up-regulates β-catenin signaling pathway and promotes transcriptional activation of c-Myc/Cyclin D1. (A) PCA was conducted to illustrate a distinct profile of circIGF1R overexpression in CMs compared to CMs treated with Ad5:cTNT-CON. (B) The volcano plot resulting from the quantitative transcriptomic analysis displays up-regulated (red) and down-regulated (blue) genes. Genes were considered significant if fold change >1.5 or <0.67, and FDR <0.05. (C) Cluster analysis and heatmap of DEGs. (D) Significant enriched GO biological process related to cardiac regeneration. (E) Significant enriched GO biological process related to Wnt signaling pathway. (F) Heatmap of DEGs associated with Wnt signaling pathway. (G) qRT-PCR assays determining the relative mRNA expression levels of β-catenin, cyclin D1, and c-Myc after circIGF1R overexpression.* n* = 3 in each group. (H and I) Relative expression of DDX5 and key proteins of β-catenin signaling pathway determined by Western blot in P1 NMCMs transfected with Ad5: cTNT-CON and Ad5: cTNT-circIGF1R, *n* = 3 in each group. (J and K) Relative expression of DDX5 and key proteins of β-catenin signaling pathway determined by Western blot in P1 NMCMs transfected with Ad5: cTNT-CON, Ad5: cTNT-circIGF1R, and Ad5: cTNT-circIGF1R + Ad5: cTNT-shDDX5, *n* = 3 in each group. (L) Graphical representation of 3-dimensional structures of the DDX5–β-catenin complex. Green: DDX5; purple: β-catenin. (M and N) Co-IP assays were carried out to confirm the interaction between DDX5 and β-catenin in P1 NMCMs. (O and P) CHIP assay to analyze the enrichment of cyclin D1 and c-Myc by β-catenin antibody, *n* = 6 in each group. (Q) CHIP assay to analyze the enrichment of cyclin D1 and c-Myc in P1 NMCMs transfected with Ad5: cTNT-CON and Ad5: cTNT-circIGF1R, *n* = 6 in each group. (R) TOP/FOP flash reporter assay in P1 NMCMs transfected with Ad5: cTNT-CON and Ad5: cTNT-circIGF1R, *n* = 6 in each group. Data are presented as mean ± SEM. NS, no significance. *P ≤ 0.05, **P ≤ 0.01, and ***P ≤ 0.001.

In the progression of CVDs, upstream regulatory genes trigger signal transduction cascades that modulate cellular phenotypes. To investigate the activity of downstream molecules and alterations in signaling pathways, we performed transcriptomics and bioinformatics analysis on cardiomyocytes overexpressing circIGF1R. Principal component analysis (PCA) identified unique features in circIGF1R-overexpressing cardiomyocytes in contrast to those treated with Ad5: cTNT-CON (Fig. 7A). Subsequent analysis revealed 4,313 differentially expressed genes (DEGs) between the circIGF1R and control groups (fold change [FC] > 1.5 or < 0.67, false discovery rate-adjusted *P* [FDR] < 0.05), visualized through volcano plots and heatmaps (Fig. 7B and C and Table S5). Gene Ontology (GO) analysis indicated significant enrichment of GO terms related to cell proliferation, including “positive regulation of DNA replication” and “cardiac muscle cell proliferation” (Fig. 7D). Notably, β-catenin signaling has been recognized as a pivotal pathway influencing a spectrum of cellular processes, including cardiomyocyte proliferation, angiogenesis, anti-apoptotic responses, and cardiac remodeling. While DDX5 is known to activate β-catenin signaling through directly interacting with β-catenin, its impact in cardiomyocytes remains unexplored [31–33]. Therefore, we further investigated the alterations in the Wnt/β-catenin signaling pathway following the overexpression of circIGF1R. The results of GO analysis revealed a significant enrichment of Wnt/β-catenin pathway-related GO terms in Fig. 7E. Next, we identified the expression of genes within the Wnt/β-catenin pathway and found that cyclin D1 (CCND1, FC = 2.42, FDR < 0.001) and c-Myc (MYC, FC = 14.39, FDR < 0.001) were markedly up-regulated (Fig. 7F). Based on these results, we hypothesize that circIGF1R might partly exert its effects via the Wnt/β-catenin pathway. To validate this hypothesis, we overexpressed circIGF1R in NMCM using Ad5: cTNT-circIGF1R and assessed β-catenin signaling activity via qRT-PCR and Western blot. The findings indicated that circIGF1R significantly enhanced the transcription levels of cyclin D1 and c-Myc, but did not affect β-catenin. However, it did increase the protein levels of β-catenin, cyclin D1, c-Myc, and DDX5 (Fig. 7G to I). To elucidate DDX5’s contribution to β-catenin signaling activation by circIGF1R, NMCMs were co-transfected with Ad5: cTNT-circIGF1R and Ad5: cTNT-shDDX5. Subsequent Western blot analysis indicated that DDX5 knockdown attenuated β-catenin pathway activation associated with circIGF1R overexpression, as shown in Fig. 7J and K. Additionally, protein docking predictions and Co-IP experiments were conducted to verify the physical interaction between DDX5 and β-catenin (Fig. 7L to N). Furthermore, to explore whether circIGF1R can up-regulate β-catenin-mediated transcriptional activity, we used TOPFlash and FOPFlash luciferase reporter assays and chromatin immunoprecipitation (ChIP) assay. The results showed that overexpression of circIGF1R promoted the binding of β-catenin to cyclin D1 and c-Myc promoter regions (Fig. 7O to Q) and enhanced the transcriptional activity of TOP/FOP (Fig. 7R). In conclusion, these findings suggest that circIGF1R promotes the transcriptional activation of cyclin D1 and c-Myc by increasing β-catenin protein levels, rather than its mRNA levels.

### Blocking the DDX5 binding abolishes the role of circIGFlR in promoting cardiomyocyte proliferation and cardiac repair after MI

To investigate the potential impact of the interaction between circIGF1R and DDX5 on cellular functions, we transfected NMCMs with Ad5: cTNT-circIGF1R alone or in combination with Ad5: cTNT-shDDX5. Immunofluorescence staining demonstrated that DDX5 inhibition markedly attenuated the efficacy of circIGF1R in promoting cardiomyocyte cell cycle re-entry and preventing apoptosis (Fig. S8A to H). Flow cytometry results supported these findings (Fig. S8I and J), indicating that DDX5 knockdown partially reverses the effects of circIGF1R on cardiomyocyte proliferation. These findings provide preliminary evidence that the interaction between circIGF1R and DDX5 is essential for regulating CM proliferation and survival.

To further explore the dynamics of the circIGF1R-DDX5 axis in vivo, we administered AAV9: cTNT-circIGF1R alone or in conjunction with AAV9: cTNT-shDDX5 directly into the peri-infarct region of mice subjected to MI (Fig. S9A). Evaluation at 28 days post-infarction revealed significant improvement in cardiac function due to circIGF1R overexpression. However, this improvement was diminished in the cohort receiving simultaneous DDX5 knockdown, as evidenced by reduced EF and FS values relative to mice overexpressing circIGF1R alone (Fig. S9B and C). Histological analyses employing Masson’s trichrome and Sirius red staining further corroborated that DDX5 silencing attenuated the positive effects of circIGF1R overexpression on scar size post-MI (Fig. S9D to F). Immunofluorescent staining showed that the anti-apoptotic effect induced by circIGF1R overexpression was partially reversed by DDX5 knockdown (Fig. S9G and H). These findings illustrate that circIGF1R activates CM cell cycle and reduces apoptosis through its interaction with DDX5, ultimately triggering the β-catenin signaling cascade.

**Fig. 8.**
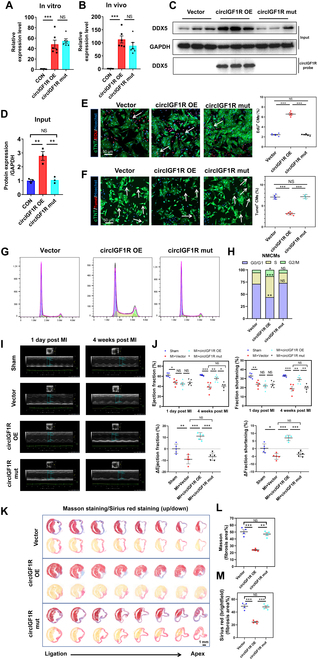
Blocking the DDX5 binding abolishes the role of circIGFlR in promoting cardiomyocytes proliferation and cardiac repair after MI. (A and B) Relative expression of circIGF1R in P1 NMCMs transfected with Ad5: cTNT-CON, Ad5: cTNT-circIGF1R, and Ad5: cTNT-circIGF1R mut and MI hearts transfected with AAV9: cTNT-CON, AAV9: cTNT-circIGF1R, and AAV9: cTNT-circIGF1R mut determined by qRT-PCR targeting the back-splicing site of circIGF1R, *n* = 6 in each group. (C and D) Relative expression of DDX5 protein determined by RNA pull-down and Western blot in P1 NMCMs transfected with Ad5: cTNT-CON, Ad5: cTNT-circIGF1R, and Ad5: cTNT-circIGF1R mut, *n* = 3 in each group. (E) Representative pictures and quantification analysis of CM proliferation quantified by immunofluorescence for DNA synthesis (EdU) in P1 NMCMs transfected with Ad5: cTNT-CON, Ad5: cTNT-circIGF1R, and Ad5: cTNT-circIGF1R mut, *n* = 6 in each group. Scale bar, 50 μm. (F) Representative pictures and quantification analysis of CM apoptosis quantified by TUNEL staining in P1 NMCMs transfected with Ad5: cTNT-CON, Ad5: cTNT-circIGF1R, and Ad5: cTNT-circIGF1R mut after OGD, *n* = 6 in each group. Scale bar, 50 μm. (G and H) Cell flow cytometry was performed to detect the cell cycle of P1 NMCMs transfected with Ad5: cTNT-CON, Ad5: cTNT-circIGF1R, and Ad5: cTNT-circIGF1R mut, *n* = 3 in each group. (I and J) Cardiac function of ejection fraction and fractional shortening among the sham group, vector group, circIGF1R group, and circIGF1R mut group at 1 and 28 day post-operation were detected by echocardiography, *n* = 5 in each group. (K to M) Masson staining and Sirius red staining were used to determine scar formation among the AAV9: cTNT-CON, AAV9: cTNT-circIGF1R, and AAV9: cTNT-circIGF1R mut at 28 dpi, *n* = 4 in each group. Scale bar, 1 mm. Data are presented as mean ± SEM. NS, no significance. *P ≤ 0.05, **P ≤ 0.01, and ***P ≤ 0.001.

To further confirm the role of DDX5 in circIGF1R-mediated effects, we mutated the circIGF1R binding site “151 to 202 nt” (termed circIGF1R-mut) to disrupt the interaction between circIGF1R with DDX5. We found that truncation of the binding site did not affect the circularization of circIGF1R (Fig. 8A and B). In vitro, NMCMs were transfected with Ad5: cTNT-circIGF1R or Ad5: cTNT-circIGF1R-mut. CircRNA pull-down and Western blot analysis indicated that mutation of the circIGF1R binding site abolished the interaction between circIGF1R and DDX5 (Fig. 8C and D). In vitro, immunofluorescence staining and flow cytometry results demonstrated that, compared to circIGF1R, overexpression of circIGF1R-mut did not promote cell proliferation, alter the cell cycle, or inhibit apoptosis (Fig. 8E to H). In vivo, cardiac function was assessed in MI mice injected with circIGF1R-mut. The results showed that overexpression of circIGF1R-mut did not confer the same therapeutic benefits as circIGF1R overexpression. Cardiac function did not improve compared to the MI-only group (Fig. 8I and J). Masson’s trichrome and Sirius red staining indicated no reduction in collagen deposition in hearts injected with circIGF1R-mut (Fig. 8K to M). In summary, disrupting the interaction between circIGF1R and DDX5 completely abolished the regenerative and reparative effects of circIGF1R on the myocardium. These findings underscore the crucial role of DDX5 in the biological function of circIGF1R.

## Discussion

Following the development of small-molecule drugs and antibody/protein drugs, nucleic acid drugs have ushered in the third wave of drug revolution. RNA-based vaccines have played a crucial role in combating the COVID-19 pandemic over recent years. However, the inherent instability of RNA in its linear form limits its broader therapeutic applications, although this instability is less problematic for vaccines [34]. To overcome this challenge, circRNAs have garnered attention for their capacity to extend RNA stability [31,35]. CircRNA is emerging as a vital regulator in various diseases, with its role in CVDs receiving increasing scrutiny [32,33,36,37]. The study of circRNA in heart regeneration is currently inadequate [20,38]. Our investigation has revealed that circIGF1R can facilitate myocardial renewal and cardiac repair while concurrently reducing cardiomyocyte apoptosis triggered by ischemia and hypoxia. Mechanistically, circIGF1R interacts directly with the DDX5 protein to activate the β-catenin signaling pathway. These insights suggest that the circIGF1R/DDX5/β-catenin axis could prove advantageous in the pursuit of advancing MI treatment and prognosis.

In this study, we initially identified circIGF1R in cardiomyocytes by analyzing a circRNA database based on cardiac regeneration capabilities. We found that circIGF1R was highly expressed during the regeneration window and was reactivated following MI or AR. Furthermore, the plasma of AMI patients showed significant elevated levels of circIGF1R in comparison to those of healthy individuals. Functional assays in mice showed that overexpressing circIGF1R markedly augmented the ratio of proliferative CMs and inhibited apoptosis. Overexpression of circIGF1R also enhanced hiPSC-CM proliferation. Additionally, our research sheds light on the function of circIGF1R in myocardial restoration, illustrating its potential to diminish scar tissue and augment cardiac function in adult mice following MI.

Next, we explored the mechanisms by which circIGF1R contributes to cardiac regeneration, expanding upon established insights into the capacity of circRNAs to serve as miRNA sponges, bind with proteins, or regulate proteins or short peptide translation. Prior research has demonstrated the ability of circIGF1R to act as a miRNA sponge [39–41]. Our investigation focused on circIGF1R’s capacity to directly interact with proteins, revealing that circIGF1R can serve as a protein scaffold, specifically interacting with DDX5 and influencing downstream gene expression.

DDX5, also called P68, is a pivotal ATP-dependent RNA helicase involved in numerous biological processes, including cell growth, DNA repair, and programmed cell death [42–44]. As a multifunctional protein influencing diverse biological behaviors, DDX5 holds important potential as a cancer biomarker or therapeutic target. Yet, its role in CVDs, especially myocardial regeneration, is not well-established. Our study observed a high expression of DDX5 in neonatal hearts and a reduction in adulthood. We also detected higher DDX5 levels in the plasma of AMI patients. In vitro, functional assays demonstrated that DDX5 overexpression promoted cardiomyocyte proliferation and inhibited apoptosis, while DDX5 silencing partially reversed the pro-proliferation and anti-apoptotic effects induced by circIGF1R. In vivo, experiments corroborated the above findings. Further, we predicted and identified the binding sites between circIGF1R and DDX5. Upon disrupting their interaction, circIGF1R’s ability to promote cardiac repair after MI was completely abolished. In conclusion, we revealed the role of the circIGF1R-DDX5 axis in cardiac regeneration regulation for the first time.

The elucidation of circIGF1R-DDX5’s biological roles prompts an inquiry into whether circIGF1R initiates signaling cascades through established pathways. The β-catenin signaling pathway, crucial for cardiac regeneration, modulates the stability and nuclear distribution of β-catenin [45–47]. Previous research in oncology has demonstrated that DDX5 modulates cell proliferation and apoptosis via this pathway [48,49], suggesting a dual mechanism: in the cytoplasm, DDX5 potentially stabilizes β-catenin by preventing its degradation within the APC/axin/GSK-3β complex; in the nucleus, it may enhance β-catenin’s transcriptional activity [46,50] In this study, our focus centered on elucidating DDX5’s cytoplasmic function. Despite its known role in regulating RNA splicing, knocking down DDX5 in cardiomyocytes did not impact circIGF1R expression. This suggests that DDX5 may not be directly involved in circIGF1R biosynthesis. Our findings reveal that circIGF1R overexpression notably elevates expression of DDX5 and activates the β-catenin pathway, which ultimately leads to transcriptional activation of cyclin D1 and c-Myc. Particularly in cardiomyocytes, we identified the interaction between DDX5 and β-catenin, a previously unreported finding. Nonetheless, the specific regulatory mechanisms and downstream effects warrant further exploration to fully comprehend the implications of these interactions.

CircRNA technology is emerging as a potential breakthrough in the next generation of RNA therapies. Endogenous circRNA can be utilized as a new drug target and a biomarker for diagnosing disease, while artificially synthesized circRNA can specifically interact with various biological targets and exert cellular effects. In this study, we identified circIGF1R as a potential target for MI treatment. However, our findings are preliminary and necessitate further comprehensive research. A primary limitation is that current research on circIGF1R is predominantly conducted in rodents. Several experiments have been performed on hiPSC-CMs and plasma from AMI patients, but they cannot accurately replicate real pathological conditions. Moreover, the detection of circIGF1R and DDX5 in AMI patients and its validity as a biomarker need to be further confirmed. In addition, this study predominantly employed virus (adenovirus/adeno-associated virus) as vectors to deliver circIGF1R for MI heart treatment, raising concerns about the biological safety of these viruses in long-term treatment for MI and potentially limiting clinical application. With advancements of in vitro circularization techniques, using extracellular vesicles or nanomaterials to deliver synthesized circRNA to specific targets may offer a safer and more efficacious approach in the future.

To summarize, we identified a cardioprotective circRNA, circIGF1R, and delineated its pivotal role in improving the prognosis of MI and facilitating cardiac function recovery. CircIGF1R initiates the activation of β-catenin signaling pathway by the interaction with DDX5, which subsequently facilitates heart regeneration and reduces myocardial apoptosis. These insights contribute a novel understanding of circRNA’s role in cardiac regeneration, presenting potential therapeutic strategies for MI.

## Methods

The datasets, methodologies, and materials generated in this study are accessible to fellow academics upon appropriate inquiry. Detailed methods are outlined in the Supplemental Materials.

### Animal model establishment

The Institutional Animal Care and Use Committee at Nanjing Medical University granted approval for the animal experiments in this study. The mice used in the experiments were obtained from Nanjing Medical University’s Animal Core Facility. They were housed in a controlled, pathogen-free environment. The MI and AR models were established in 8-week-old or 1-day-old ICR mice, respectively, following methodologies previously published [6,51].

### Animal model construction and intervention

#### Myocardial infarction model in adult mice

Adult ICR mice, aged 8 weeks, were anesthetized intraperitoneally with 1.2% Avertin. Subsequent steps included tracheal intubation and mechanical ventilation at a respiratory rate of 120 to 140 breaths per minute. The left anterior descending (LAD) artery was identified through a surgical incision in the third intercostal space, exposing the heart. Ligation of the LAD was achieved 2 to 3 mm distal to the left atrial appendage using a 7-0 unabsorbable suture, with successful coronary artery occlusion confirmed by the pallor of the distal myocardium. For the local injection of AAV9: cTNT-circIGF1R, AAV9: cTNT-circIGF1R mut, AAV9: cTNT-shDDX5, and AAV9: cTNT-CON into the infarct border zone, a microsyringe equipped with a 33G needle was employed. The viral dose administered per mouse was 1 × 10^9^ viral genomes (v.g.). These adeno-associated viruses were supplied by Genechem Company (Shanghai, China), each boasting a titer of 1 × 10^13^ v.g./ml. After injection, closure was accomplished with absorbable sutures for the rib and muscle layers and 6-0 nylon sutures for the skin. Animals were extubated upon regaining consciousness.

#### Apical resection model in neonatal mice

An ice bed was utilized to anesthetize neonatal ICR mice through hypothermia. The skin was incised and the intercostal muscles were meticulously dissected, leading to a lateral thoracotomy at the third intercostal space. Approximately 15% of the left ventricular apex was excised carefully to avoid damage to the endocardium. We employed a 36G microsyringe needle for the injection of Ad5: cTNT-sicircIGF1R and Ad5: cTNT-CON around the apex of the P1 heart. The administered viral dosage totaled 2 × 10^7^ pfu per mouse. These viruses were sourced from Genechem Company (Shanghai, China), with each adenovirus boasting a titer of 2 × 10^10^ v.g./ml. Post-procedure, animals were removed from the ice, and the thorax was closed with 7-0 non-absorbable sutures, followed by the application of skin adhesive for the skin incision. Sham-operated controls underwent identical procedures excluding the AR.

#### Intraperitoneal injection model in neonatal mice

In P1 mice, AAV9: cTNT-circIGF1R and AAV9: cTNT-CON solution were diluted fivefold with phosphate-buffered saline (PBS) before intraperitoneal injection. The administered viral dosage totaled 1 × 10^11^ pfu per mouse.

### Echocardiographic evaluation

We assessed cardiac function in mice using the VisualSonics Vevo 2100 imaging system. Echocardiographic evaluations were performed at predetermined time points: 24 h and 28 days post-MI induction, and 24 h and 22 days after AR. The mice were put under anesthesia using 1% to 2% isoflurane. Positioned on a warm platform, a high-resolution (40 MHz) ultrasound probe, coupled with warmed gel, facilitated detailed cardiac imaging through 2-dimensional transthoracic echocardiography. Utilizing this imaging technique, precise calculations of left ventricular end-diastolic volumes and end-systolic volumes, as well as wall thicknesses and interventricular septal thicknesses in both diastole and systole were achieved. Vevo LAB software was employed to compute key functional parameters, namely, left ventricular ejection fraction and shortening fraction. Operators, blinded to the treatment groups, ensured unbiased data collection, reinforcing the objectivity of our cardiac function analysis following intervention.

### Collection of clinical plasma samples

Between January 2021 and June 2023, we procured plasma samples from 30 healthy individuals and 30 patients with AMI, as diagnosed at Jiangsu Province Hospital, in adherence to the criteria set forth by the Fourth Universal Definition of Myocardial Infarction (2018). The exclusion criteria were comprehensive, excluding participants with prosthetic heart valves, recent cardiac stent placements or cardiac revascularization within the previous year, myocarditis, uncontrolled severe arrhythmias, aortic aneurysm or carotid artery dissection, advanced hepatic and renal disorders, significant hematological or infectious diseases, severe cognitive impairments, autoimmune disorders, major respiratory diseases, thyroid dysfunctions, and active cancer. The ethical standards of the study were upheld with approval from the local ethics committee (approval no. 2022-NT-02). Prior to the inclusion, all individuals were duly informed and provided their consent.

### Isolation and neonatal cardiomyocyte culture

We isolated heart cells from very young mice (just born or 1 day old) and followed a set process to clean and break down the heart tissue into individual cells. This process involved washing the hearts in a saline solution, then using a special mix of enzymes to gently separate the cells until we had a mixture of single cells. Next, we used a spinning technique (centrifugation) to sort the cells based on their type. Cardiomyocytes, or heart muscle cells, were carefully collected from a specific layer of the cell mixture. This allowed us to remove other cell types, like fibroblasts and blood cells, that we did not need. The heart cells we kept were then placed onto dishes that had a special coating (fibronectin) to help the cells attach and grow. We aimed for a certain number of cells per dish or per well in a plate, providing them with a special nutrient mix made just for young heart cells. This setup helps us study how these heart cells grow and react in a controlled environment, similar to conditions inside the body.

To investigate the functional impact of circIGF1R and DDX5 on CMs, the cells were subjected to transfection with the following adenoviral vectors: Ad5: cTNT-circIGF1R (multiplicity of infection [MOI] = 100), Ad5: cTNT-circIGF1R mut (MOI = 100), Ad5: cTNT-sicircIGF1R (MOI = 100), Ad5: cTNT-DDX5 (MOI = 50), or Ad5: cTNT-DDX5i (MOI = 50) for a duration of 24 h.

### HiPSC-CM differentiation and culture

HiPSC-CMs were obtained from Nanjing Elp Regenerative Medicine Technology Co., Ltd. The culture and differentiation process was as follows: undifferentiated hiPSCs were cultured to 90% confluence and treated with #1 medium (RPMI 1640, B27 supplement minus insulin, and 2-Phospho-L-ascorbic acid trisodium salt) with 6 μM CHIR-99021 on days 0 and 1. On day 2, the medium was switched to #1 medium only, followed by #1 medium with 5 μM IWR-1 on days 3 and 4. From day 5 to day 8, the cells were maintained in #1 medium, then switched to #2 medium (RPMI 1640, B27 supplement, and 2-Phospho-L-ascorbic acid trisodium salt) every other day. At day 30 post-differentiation, purified hiPSC-CMs were thawed for proliferation analysis. A total of 1 × 10^5^ cells were seeded in a 24-well plate, allowed to adhere for 48 h, and maintained with fresh medium every other day. The cells were treated with Ad5: cTNT-circIGF1R (MOI = 100) for 24 h before being fixed and stained for analysis.

### Histological evaluation

Following echocardiographic assessment, the morphological examination of murine cardiac tissue was conducted employing Masson’s trichrome and Sirius red stains. Dissected hearts were immediately fixed in a 4% paraformaldehyde solution to preserve tissue integrity. Subsequent to fixation, tissues were processed for paraffin embedding, ensuring the preservation of cellular structures for histological analysis. Serial sections of 5 μm thickness were generated spanning from the apex to the base of the heart, providing comprehensive coverage for subsequent evaluation. The prepared sections underwent staining with Masson’s trichrome to delineate cardiac muscle fibers and with Sirius red to highlight fibrous collagen deposits, facilitating a detailed assessment of cardiac architecture and the quantification of fibrotic tissue. Utilization of ImageJ software allowed for precise measurement of the fibrotic area, affording an objective comparison of myocardial fibrosis across samples.

### Immunofluorescent staining

Heart tissue sections, embedded in paraffin, underwent deparaffinization and rehydration, followed by antigen retrieval using a citrate-EDTA buffer facilitated by heat induction. Cultured cardiomyocytes adhered to the substrate were cleansed with PBS and then fixed for 20 min with a 4% paraformaldehyde solution. 0.1% Triton X-100 was used for half an hour to facilitate permeabilization. Post-permeabilization, samples underwent a PBS wash, treated with a 5% bovine serum albumin solution in PBS for 2 h at ambient temperature, and exposed to specific primary antibodies for an entire night at 4°C. After incubation with primary antibody, samples were rinsed 3 times with PBS (10 min per rinse), followed by a 2-h incubation with secondary antibodies labeled by fluorescence, and staining nuclei with Hoechst 33342 (Thermo Fisher, H3570) for 20 min. Observations were conducted using a confocal microscope (Zeiss, LSM800). All details regarding antibodies can be found in Table S3.

### 5-Ethynyl-2′-deoxyuridine staining

To evaluate cardiomyocyte proliferation, we utilized 5-Ethynyl-2′-deoxyuridine (EdU) incorporation. The cardiomyocytes were exposed to an EdU solution (10 μM in Dulbecco's modified Eagle medium [DMEM] with 10% fetal bovine serum [FBS]) and were later fixed with 4% paraformaldehyde. For in vivo studies, mice were injected with EdU intraperitoneally (150 to 200 mg/kg) 48 h before euthanasia, after which hearts were harvested and processed into paraffin sections for antigen retrieval. Both fixed cardiomyocytes and tissue sections were permeabilized, blocked, and exposed to Click Additive Solution for 30 min in the dark to visualize EdU incorporation. Immunofluorescence staining was then applied as needed, with confocal microscopy (Zeiss, LSM800) used to image and analyze proliferative activity in the samples.

### Flow cytometry analysis

Trypsin (0.25%) was utilized to isolate primary NMCMs into single cells. The enzymatic reaction was quenched by DMEM containing 10% FBS. Afterward, the cells were pelleted through centrifugation and the supernatant was discarded. To ensure complete removal of residual FBS and trypsin, the cell pellet was washed repeatedly with chilled PBS. The cells were then treated with cold 70% ethanol and left to fix for the entire night at 4°C. Following fixation, cells were cleaned with cold PBS and centrifuged to remove the supernatant. After resuspending the cell pellet in propidium iodide/RNase Staining Buffer (BD Biosciences), it was incubated in a water bath at 37°C for 40 min to ensure complete staining. Flow cytometry analysis was utilized to assess the cell cycle distribution of the stained cells. Flow cytometric data acquisition was performed, and the collected data were evaluated by FlowJo software from BD Biosciences.

### RNA isolation and qRT-PCR analysis

TRIzol Reagent (Invitrogen) was used to extract total RNA from both neonatal and adult mouse hearts. We assessed the RNA integrity and purity with a NanoDrop spectrophotometer, considering *A*_260_/*A*_280_ ratios within the optimal range of 2.0 ± 0.10 as indicative of purity for further analysis. Following extraction, the process of reverse-transcribing RNA into cDNA was carried out using HiScript III RT SuperMix. We performed qRT-PCR using the cDNA generated as a template, and conducting it with SYBR Green (Vazyme Biotech, Q131-02) on the QuantStudio 7 system (ABI). The 2^−ΔΔCt^ method was carried out to determine the relative expression levels of targeted genes. The primer sequences are listed in Table S1.

### Western blot analysis

Total protein extraction from myocardium and primary cardiomyocytes was performed with radioimmunoprecipitation assay (RIPA) buffer, enriched with a cocktail of protease inhibitors, to ensure comprehensive inhibition of protease activity. The entire procedure was meticulously conducted on ice to mitigate any potential degradation of proteins. Subsequent to extraction, proteins were denatured by amalgamation with 4×SDS-PAGE loading buffer and subjected to a temperature of 95°C for 10 min to ensure complete denaturation. The denatured protein samples were then electrophoretically separated on an SDS-PAGE gel, followed by their transfer onto polyvinylidene fluoride (PVDF) membranes. In order to avoid non-specific binding, we employed 5% skim milk to block the membranes for 1 h at ambient temperature. For the purpose of protein detection, primary antibodies were used to incubate the membranes overnight at 4°C. Afterward, the membranes were carefully washed with 1× Tris-buffered saline with Tween (TBST) buffer and subsequently exposed to the corresponding horseradish peroxidase (HRP)-conjugated secondary antibodies, to enable chemiluminescent detection. The ECL was used for detection, with results displayed on a ChemiDoc imaging system from Bio-Rad. Quantitative analysis of the band intensity was conducted utilizing ImageJ software, allowing for precise measurement of protein expression levels. All details regarding antibodies can be found in Table S3.

### FISH analysis

To elucidate the intracellular distribution of circIGF1R within cardiomyocytes, we employed Cy3-labeled circIGF1R probes in our FISH analysis. This procedure was meticulously executed utilizing the FISH Kit (RiboBio, catalog C10910). The circIGF1R and 18S ribosomal RNA FISH probes, both essential for our study, were custom-designed and synthesized by RiboBio. To counterstain nuclei, we utilized Hoechst 33342 (Beyotime, C1022), which facilitates the precise demarcation of cellular nuclei against the fluorescent signal of the probes. The acquisition of fluorescent images was carried out using a confocal microscope (Zeiss, LSM800), enabling the high-resolution visualization of circIGF1R distribution in relation to the cellular architecture.

### RNA pull-down assay

HEK293T cells were transfected with the circIGF1R overexpression plasmid and subjected to RNA pull-down experiments employing the Pierce Magnetic RNA-Protein Pull-Down Kit (Thermo Fisher Scientific, 20164). RNA probes labeled with biotin were used to specifically detect circIGF1R and aid in isolating RNA–protein interactions. Subsequent to the probe hybridization, magnetic beads were introduced to the complex to proceed with immunoprecipitation. The RNA and associated proteins were then eluted from the beads. Proteins bound in the complex were subsequently isolated and identified through label-free MS and validated via Western blot analysis. Concurrently, the RNA component was subjected to qPCR and RNA-seq for comprehensive analysis.

### RIP assay

The Magna RIP RNA-Binding Protein Immunoprecipitation Kit (Millipore, 17-700) was used to perform the RIP experiment, closely following methodologies outlined in prior studies [52,53]. RIP buffer was used to lyse HEK293T cells. After centrifugation, the resulting supernatant was then mixed with magnetic beads that had been pre-coated with anti-DDX5 antibodies (Abcam, ab126730). Immunoglobulin G (IgG) served as the negative control throughout the process. Both RNAs and DDX5 proteins were meticulously isolated and purified from the immunoprecipitated complexes. Detection of the RNAs was performed via PCR, whereas the DDX5 proteins were examined through Western blot analysis.

### ChIP assay

According to the manufacturer’s protocol, ChIP assays were performed using the SimpleChIP enzymatic ChIP kit (CST, #9002). HEK293T cells transfected with circIGF1R or β-catenin-overexpressing plasmids were collected and cross-linked with 1% formaldehyde, quenched with glycine, and then sonicated to shear chromatin to an average DNA length of 500 to 1,000 bp. Chromatin fragments were incubated with IgG or anti-β-catenin antibody (Abcam, ab32597) at 4°C for 24 h, and magnetic beads were used to bind antibody target protein–DNA complexes. The eluted and purified immunoprecipitated DNA were used as templates for determining levels of c-Myc or cyclin D1 by RT-qPCR analysis and agarose electrophoresis PCR, normalized to IgG control. The c-Myc or cyclin D1 promoter primer sequences in ChIP assays were listed as follows [54]:

cyclin D1-Forward: 5′-GACTACAGGGGAGTTTTGTTG-3′

cyclin D1-Reverse: 5′-TCGGCTCTCGCTTCTGCTG-3′

c-Myc-Forward: 5′-GCTCTCCACTTGCCCCTTTTA-3′

c-Myc-Reverse: 5′-GTTCCCAATTTCTCAGCC-3′

### TOPFlash and FOPFlash luciferase reporter assay

To assess the transcriptional activity of β-catenin signaling, TOPFlash and FOPFlash assays were conducted. NMCMs with 60% to 80% confluence in 24-well plates were prepared for transfection. According to the manufacturer’s protocol, co-transfection was performed with TOPFlash plasmid (Beyotime, D2501), FOPFlash plasmid (Beyotime, D2503), and Renilla luciferase plasmid, along with circIGF1R or β-catenin overexpressing plasmids using ExFect Transfection Reagent (Vazyme, T101). In detail, TOPFlash is a luciferase reporter designed to measure β-catenin/TCF4 transcriptional activity via TCF4 binding sites. FOPFlash, with mutant TCF4 binding sites, serves as a negative control. Renilla luciferase is used as an internal control for transfection efficiency. Then, the cells were washed, harvested, and lysed 36 h after transfection. Detection of luciferase activities was conducted with the Dual Luciferase Reporter Gene Assay Kit (Beyotime, RG027). Renilla luciferase activity was used for normalization.

### Co-IP assay

Primary neonatal mice cardiomyocytes were harvested and lysed to prepare for Co-IP analysis. Overnight at 4°C, the protein lysates were exposed to primary antibodies against DDX5 (Abcam, ab126730) or β-catenin (Proteintech, 51067-2-AP). Subsequently, the protein–antibody complexes were trapped by utilizing protein G/A agarose beads (ACE Biotechnology, AM001-02) for 5 to 6 h at 4°C. Then, the complexes underwent triple washing with lysis buffer before elution and precipitation. The final protein samples underwent Western blot analysis for subsequent detection and quantification.

### Transcriptomics and data analysis

 Total RNA was extracted using the TRIzol reagent. RNA purity and concentration were measured with a NanoDrop 2000 spectrophotometer. The transcriptome library was then constructed following the VAHTS Universal V5 RNA-seq Library Preparation Kit protocol. Sequencing was performed on an Illumina NovaSeq 6000 platform, generating paired-end reads of 150 base pairs. The initial sequencing data were processed using the fastp software for quality control, and alignment to the reference genome was carried out with HISAT2. Differential gene expression analysis was performed using DESeq2, identifying genes with significant expression changes based on a *q* value threshold of <0.05 and a fold change of >1.5 or <0.67. The resulting data were further analyzed using the GO (http://www.geneontology.org/) database to identify enriched pathways and elucidate the functional implications of the DEGs. The entire sequencing process was conducted by Ouyiluming Co., Ltd. (Shanghai, China), ensuring high technical quality. Statistical significance was determined with a cutoff of *P* < 0.05.

### Statistical analysis

The data were expressed as mean ± SEM and analyzed using Prism 8 software (GraphPad). The Shapiro–Wilk test assessed the normality of data distribution. In instances where data follows a normal distribution, distinctions between 2 groups were assessed utilizing Student’s *t* test. In the comparative analysis of multiple groups, the application of one-way ANOVA supplemented by the post-hoc Tukey test was employed to ascertain statistically significant disparities among the groups under consideration. For data not conforming to normal distribution, the Kruskal–Wallis test was utilized for evaluating statistical significance. Survival rates were analyzed utilizing the Kaplan–Meier method and assessed for statistical significance by the log-rank test. The significance level was set as follows: *P ≤ 0.05, **P ≤ 0.01, and ***P ≤ 0.001.

## Data Availability

CircRNA expression profiles of adult and neonatal rats hearts were obtained from Werfel et al.’s study (PubMed ID: 27476877).
