# Supplementary material for: Circular RNA IGF1R Promotes Cardiac Repair via Activating β-Catenin Signaling by Interacting with DDX5 in Mice after Ischemic Insults
Source: Research (Wash D C). 2024 Aug 27;7:0451. doi: 10.34133/research.0451 (PMC11347128; doi:10.34133/research.0451)
Supplement: Supplementary 1 — Materials and Methods Figs. S1 to S9 Tables S1 to S5 [file research.0451.f1.zip › Supplemental material.docx]

**Circular RNA IGF1R promotes cardiac repair via activating** β**-catenin signaling by interacting with DDX5 in mice after ischemic insults**

Tian-Kai Shan ^a,#^, Tong-Tong Yang ^a,#^, Peng Jing ^a^, Yu-Lin Bao ^a^, ^,^Liu-Hua Zhou ^a^, Ting Zhu ^a^, Xin-Ying Shi ^a^, Tian-Wen Wei ^a^, Si-Bo Wang ^a^, Ling-Feng Gu ^a^, Jia-Wen Chen ^a^, Ye He ^a^, Ze-Mu Wang ^a^, Qi-Ming Wang ^a^, Li-Ping Xie ^b^, Ai-Hua Gu ^c^, Yang Zhao ^d^, Yong Ji ^b^, Hao Wang ^a,*^, and Lian-Sheng Wang ^a,*^

a Department of Cardiology, the First Affiliated Hospital of Nanjing Medical University, Nanjing, 210029, China.

b Key Laboratory of Cardiovascular and Cerebrovascular Medicine, Key Laboratory of Targeted Intervention of Cardiovascular Disease, Collaborative Innovation Center for Cardiovascular Disease Translational Medicine, Nanjing Medical University, Nanjing, China.

c State Key Laboratory of Reproductive Medicine, School of Public Health, Nanjing Medical University, Nanjing, China.

d Department of Biostatistics, School of Public Health, China International Cooperation Center for Environment and Human Health, Nanjing Medical University, Nanjing, 210029, China.

# Tian-Kai Shan, and Tong-Tong Yang made an equal contribution to this study.

*Corresponding author: Lian-Sheng Wang, MD, PhD; Department of Cardiology, the First Affiliated Hospital of Nanjing Medical University, 300 Guangzhou Road, Nanjing, 210029, Jiangsu Province, China. E-mail: drlswang@njmu.edu.cn；Fax/Tel: +86 25 83724440; Hao Wang, MD，PhD, Department of Cardiology, the First Affiliated Hospital of Nanjing Medical University, 300 Guangzhou Road, Nanjing, 210029, Jiangsu Province, China. E-mail: HaowangNJ@njmu.edu.cn.

**Figure S1**


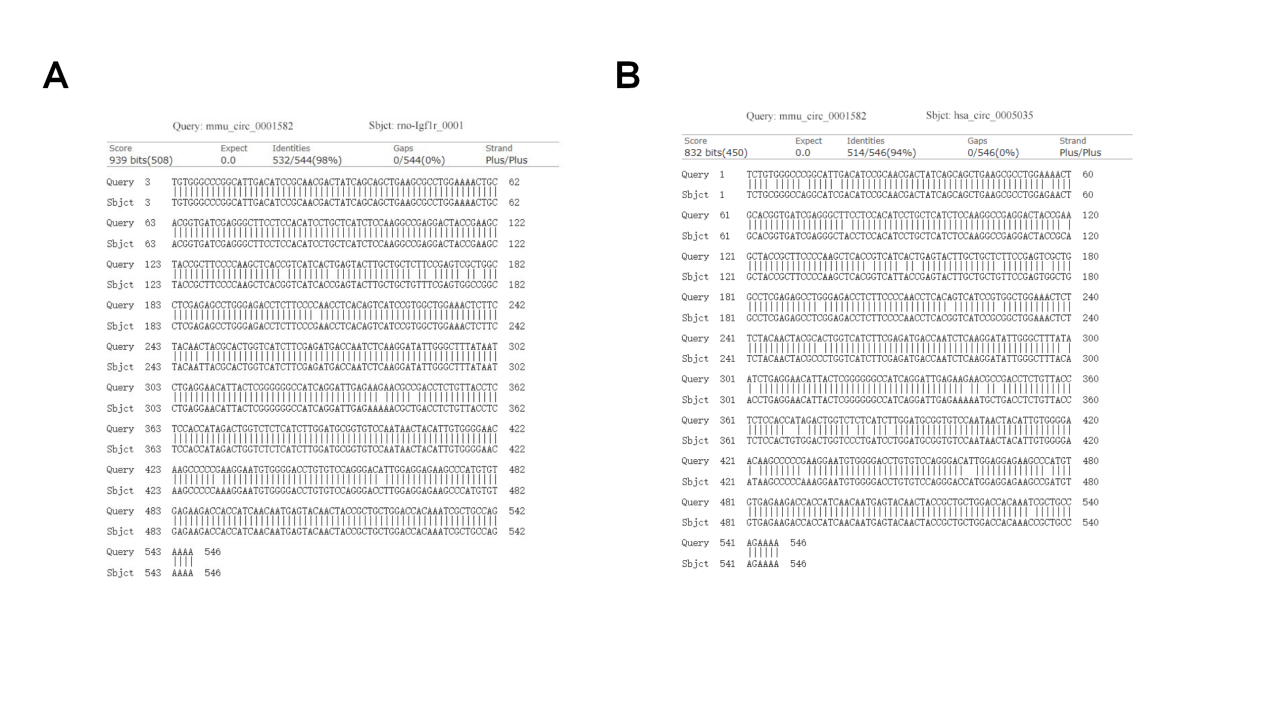


**Figure S1. circIGF1R homology analysis between rat, mouse and human.**

(A and B) Species conservation analysis of the circIGF1R sequence between rat, mouse and human using the BLAST Browser.

**Figure S2**


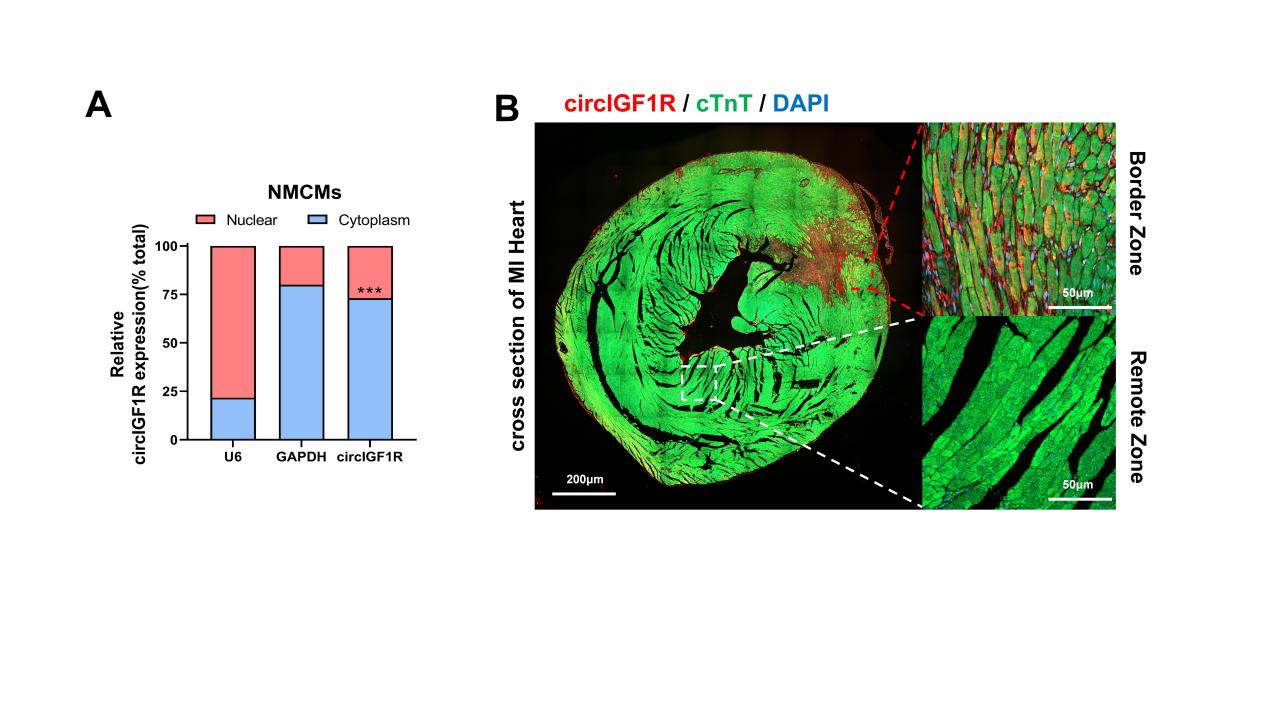


**Figure S2. Analysis of circIGF1R expression and distribution**

(A) Proportion of circIGF1R mRNA expression in the nucleus and cytoplasm of cardiomyocytes, n=3 in each group. (B) RNA-FISH assays of circIGF1R distribution in hearts of mice after myocardial infarction injury. Scale bars, 50 μm. Scale bars, 200 μm. Data are presented as mean ± SEM. ns: no significance. *P ≤0.05. **P≤0.01. ***P ≤0.001.

**Figure S3**


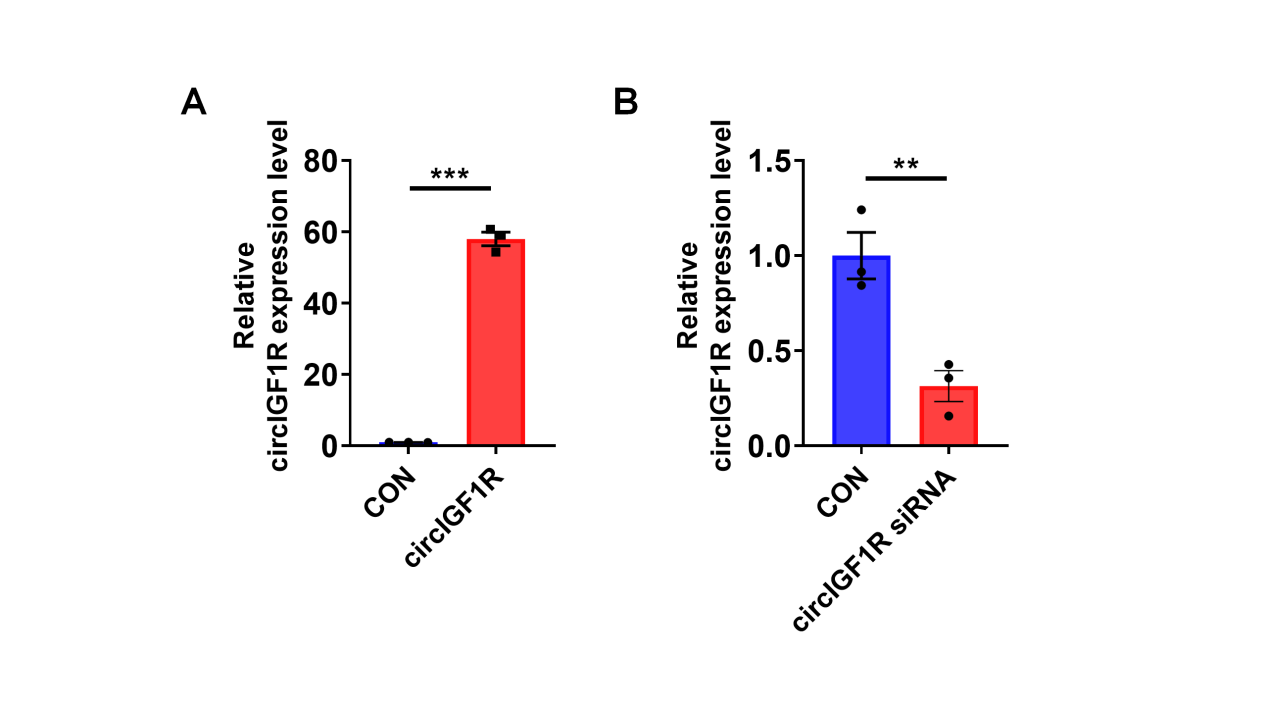


**Figure S3. circIGF1R expression analysis after overexpression and knockdown.**

(A and B) Relative expression of circIGF1R determined by qRT-PCR in NMCMs transfected with Ad5: cTNT-CON, Ad5: cTNT-circIGF1R and Ad5: cTNT-sicircIGF1R. Data are presented as mean ± SEM. ns: no significance. **P≤0.01. ***P ≤0.001.

**Figure S4**

**
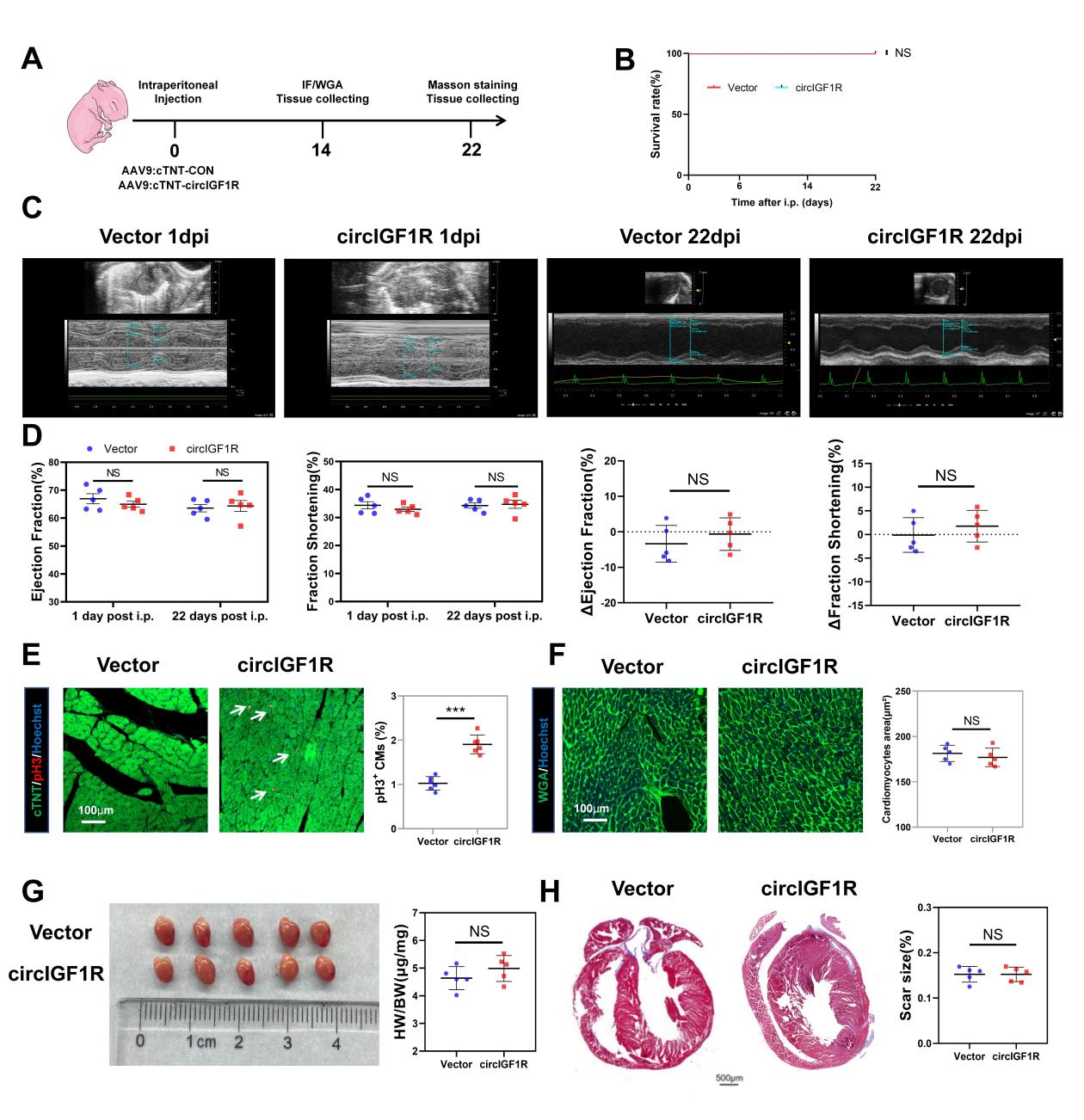
**

**Figure S4. Overexpression of circIGF1R enhances neonatal cardiomyocyte proliferation *in vivo*.**

1. Experimental pattern: AAV9: cTNT-CON or AAV9: cTNT-circIGF1R was intraperitoneally injected into P1 mice. Hearts were harvested at 14 and 22 days post injection to evaluate cardiomyocyte proliferation and scar area, respectively. Echocardiography was performed to detect cardiac function. (B) Overall survival rate in mice treated with AAV9: cTNT-CON or AAV9: cTNT-circIGF1R, n=20 in each group. (C and D) Cardiac function of ejection fraction and fractional shortening among the AAV9: cTNT-CON or AAV9: cTNT-circIGF1R treated mice at P1 and P22 were detected by echocardiography, n=5 in each group. (E) Representative pictures and quantification analysis of CM proliferation quantified by mitosis (pH3) in AAV9: cTNT-CON or AAV9: cTNT-circIGF1R groups at P14, n=6 in each group. Scale bars, 100 μm. (F) Representative pictures and quantification analysis of CM size quantified by WGA immunofluorescence in AAV9: cTNT-CON or AAV9: cTNT-circIGF1R groups at P14, n=6 in each group. Scale bars, 100 μm. (G) Heart weight/body weight (HW/BW) ratio and cardiac morphology between AAV9: cTNT-CON and AAV9: cTNT-circIGF1R at P14, n=5 in each group. (H) Masson’s trichrome staining were used to determine scar formation between the AAV9: cTNT-CON or AAV9: cTNT-circIGF1R treated mice at P22, n=5 in each group. Scale bars, 500 μm. Positive staining cardiomyocytes were indicated by arrows. Data are presented as mean ± SEM. ns: no significance. *P ≤0.05. **P≤0.01. ***P ≤0.001.

**Figure S5**


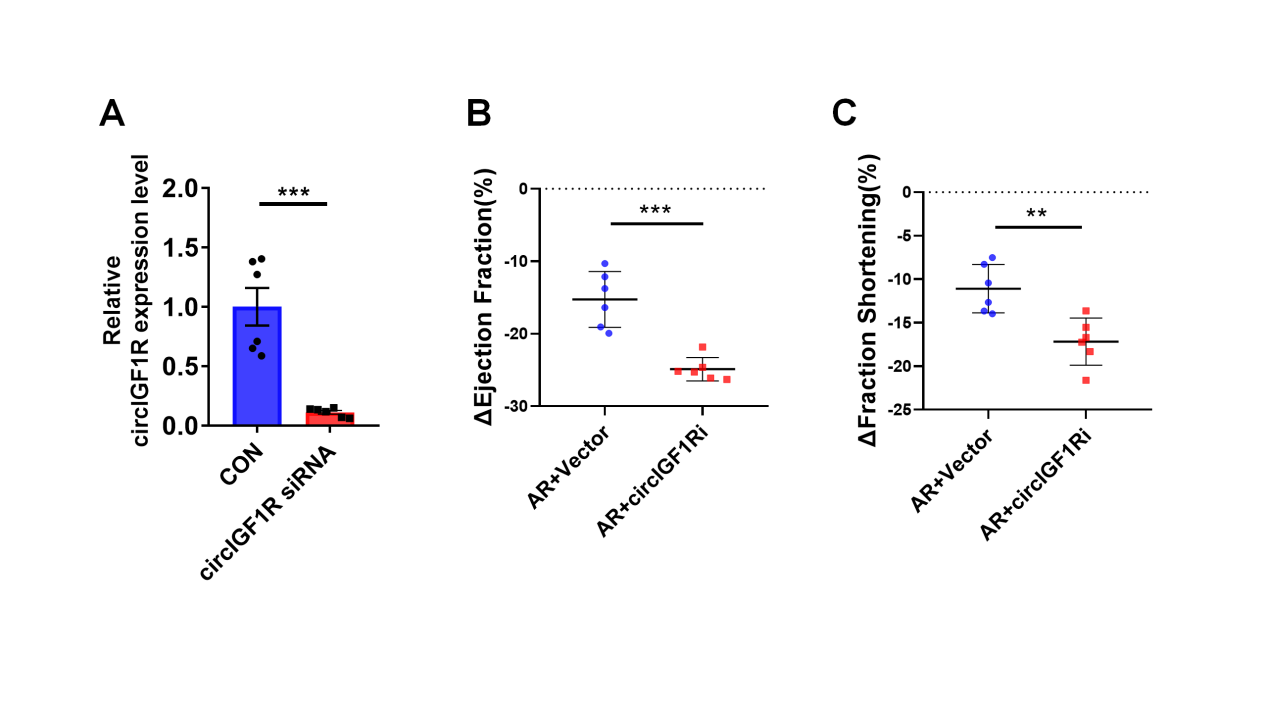


**Figure S5. Knockdown of circIGF1R suppresses cardiac function in neonatal mice after AR.**

(A) Relative expression of circIGF1R determined by qRT-PCR in vivo transfected with Ad5: cTNT-CON and Ad5: cTNT-sicircIGF1R. (B and C) Cardiac function of ejection fraction and fractional shortening among the Ad5: cTNT-CON and Ad5: cTNT-sicircIGF1R treated mice at 1 and 22 day post operation were detected by echocardiography, n=6 in each group. Data are presented as mean ± SEM. *P ≤0.05. **P≤0.01. ***P ≤0.001.

**Figure S6**


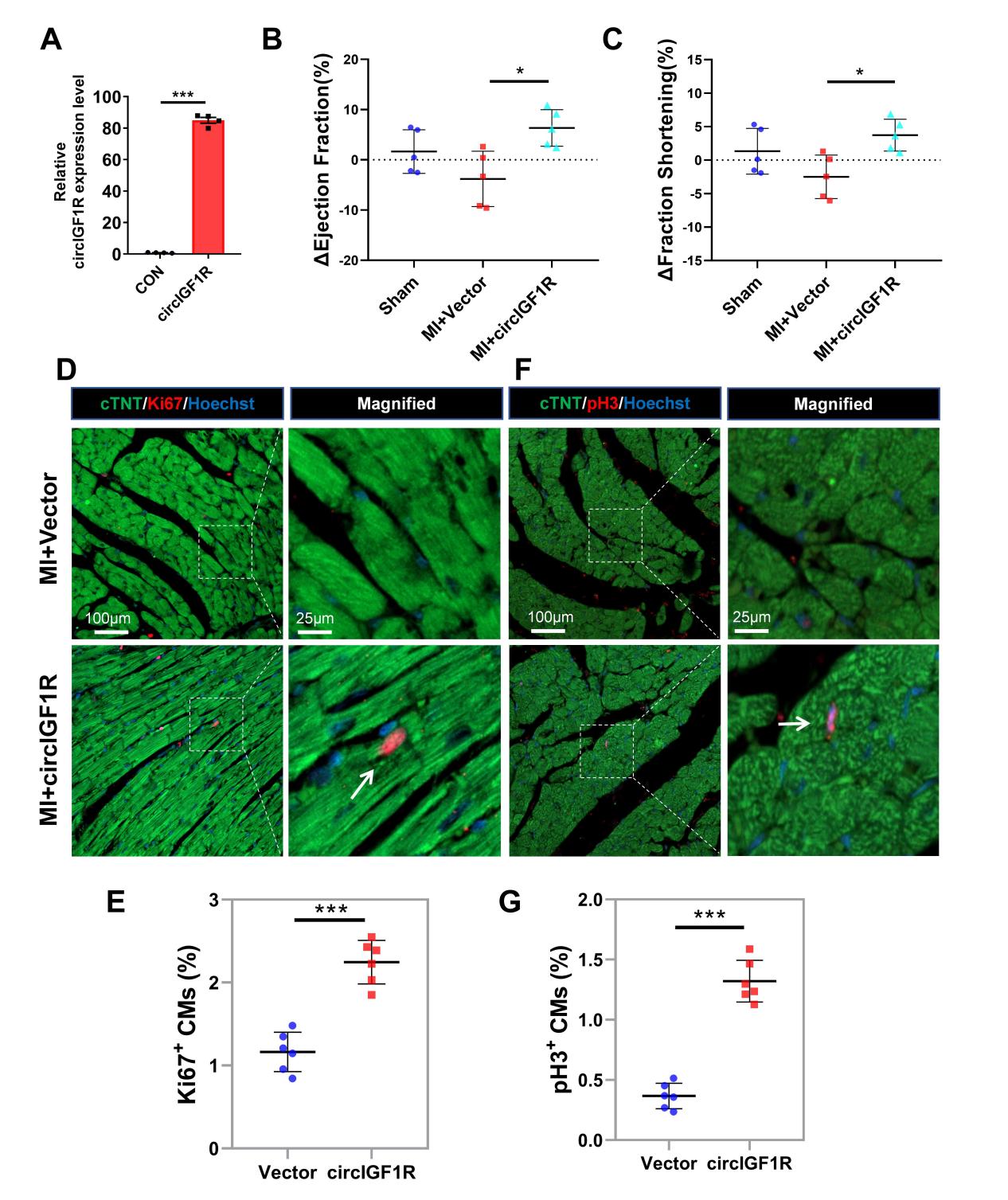


**Figure S6. Overexpression of circIGF1R promotes adult cardiac regeneration and repair after in adult mice after MI**

(A) Relative expression of circIGF1R determined by qRT-PCR in vivo transfected with AAV9: cTNT-CON and AAV9: cTNT-circIGF1R. (B and C) Cardiac function of ejection fraction and fractional shortening among the sham, AAV9: cTNT-CON or AAV9: cTNT-circIGF1R treated mice at 1 and 28 day post operation were detected by echocardiography, n=5 in each group. (D-G) Representative pictures and quantification analysis of CM proliferation quantified by cell-cycle activity (Ki67), and mitosis (pH3) in infarct border zone in AAV9: cTNT-CON or AAV9: cTNT-circIGF1R groups after MI, n=6 in each group. Scale bars, 100 μm. Scale bars, 25 μm. Picture in each rectangular box was enlarged in the neighboring right panel. Data are presented as mean ± SEM. *P ≤0.05. **P≤0.01. ***P ≤0.001.

**Figure S7**

**
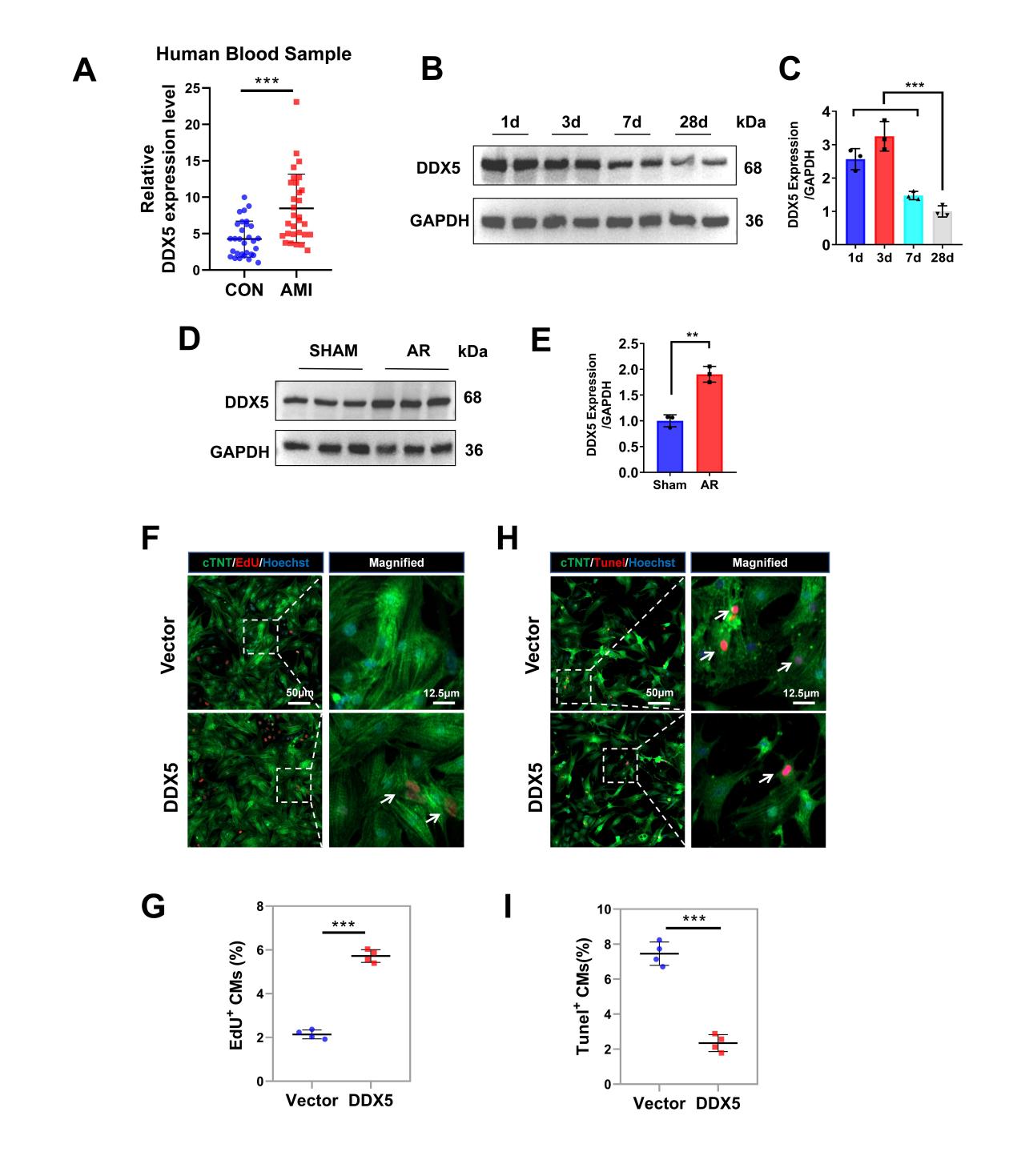
**

**Figure S7. DDX5 promotes cardiac regeneration and inhibits apoptosis.**

1. Relative expression of DDX5 mRNA determined by qRT-PCR in the plasma of patients with or without AMI within 7 days, n=30 in each group. (B and C) Relative expression of protein DDX5 determined by western blot in hearts of mice at different ages and quantification analysis, n=3 in each group. (D and E) Relative expression of protein DDX5 determined by western blot in hearts of mice after AR and quantification analysis, n=3 in each group. (F and G) Representative pictures and quantification analysis of CM proliferation quantified by immunofluorescence for DNA synthesis (EdU) in P1 NMCMs transfected with Ad5: cTNT-CON and Ad5: cTNT-DDX5, n=6 in each group. Scale bars, 50 μm. Scale bars, 12.5 μm. (H and I) Representative pictures and quantification analysis of CM apoptosis quantified by TUNEL staining in P1 NMCMs transfected with Ad5: cTNT-CON and Ad5: cTNT-DDX5 after OGD, n=6 in each group. Scale bars, 50 μm. Scale bars, 12.5 μm. Picture in each rectangular box was enlarged in the neighboring right panel. Data are presented as mean ± SEM. ns: no significance. *P ≤0.05. **P≤0.01. ***P ≤0.001.

**Figure S8**

**
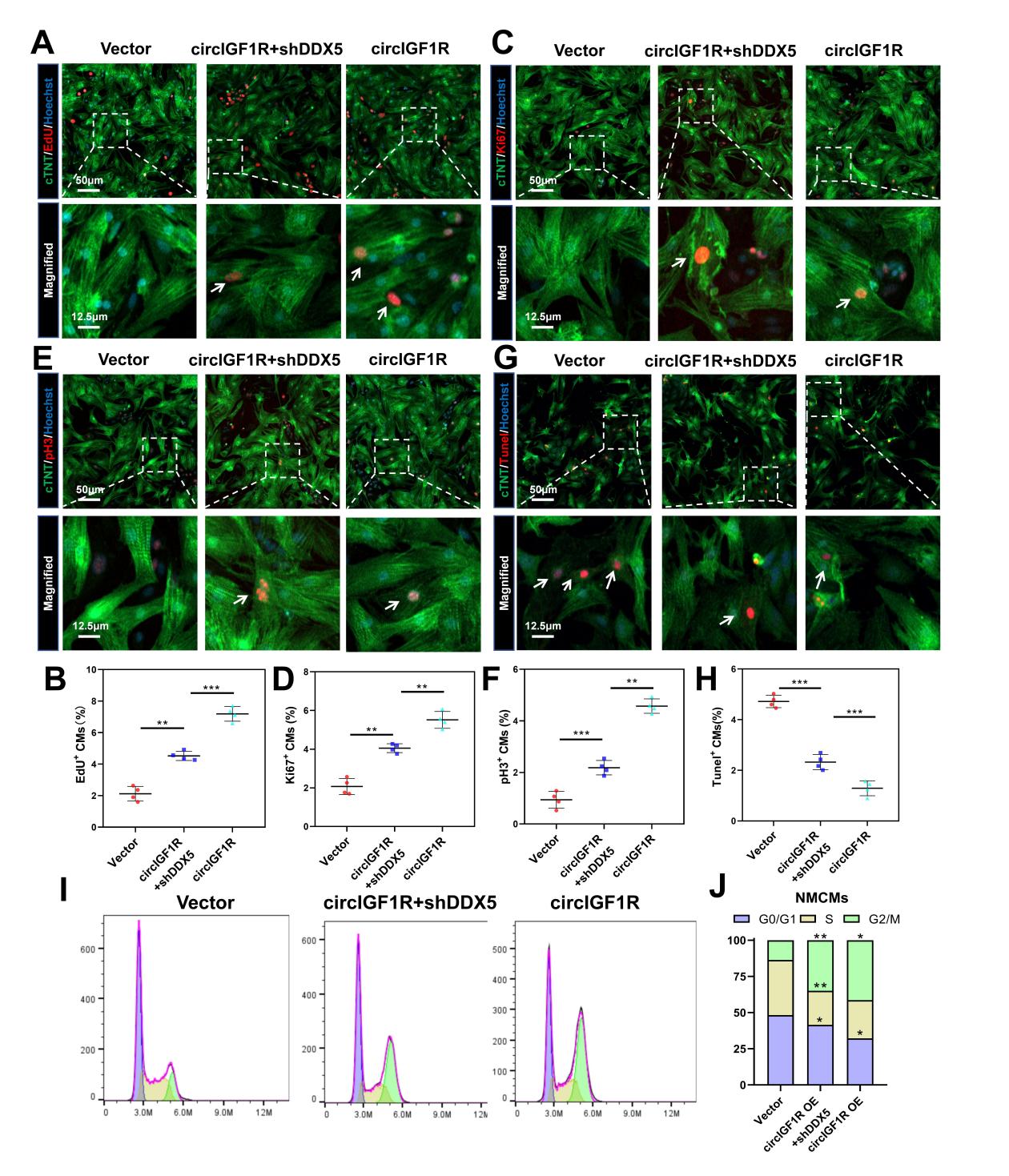
**

**Figure S8. circIGF1R promotes CMs proliferation and inhibits apoptosis through DDX5 *in vitro*.**

1. F) Representative pictures and quantification analysis of CM proliferation quantified by immunofluorescence for DNA synthesis (EdU), cell-cycle activity (Ki67), and mitosis (pH3) in P1 NMCMs transfected with Ad5: cTNT-CON, Ad5: cTNT-circIGF1R, and Ad5: cTNT-circIGF1R+Ad5: cTNT-shDDX5, n=4 in each group. Scale bars, 50 μm. Scale bars, 12.5 μm. (G and H) Representative pictures and quantification analysis of CM apoptosis quantified by TUNEL staining in P1 NMCMs transfected with Ad5: cTNT-CON, Ad5: cTNT-circIGF1R, and Ad5: cTNT-circIGF1R+Ad5: cTNT-shDDX5 after OGD, n=4 in each group. Scale bars, 50 μm. Scale bars, 12.5 μm. (I and J) Cell flow cytometry was performed to detect the cell cycle of P1 NMCMs transfected with Ad5: cTNT-CON, Ad5: cTNT-circIGF1R, and Ad5: cTNT-circIGF1R+Ad5: cTNT-shDDX5, n=3 in each group. Picture in each rectangular box was enlarged in the neighboring right panel. Data are presented as mean ± SEM. *P ≤0.05. **P≤0.01. ***P ≤0.001.

**Figure S9**

**
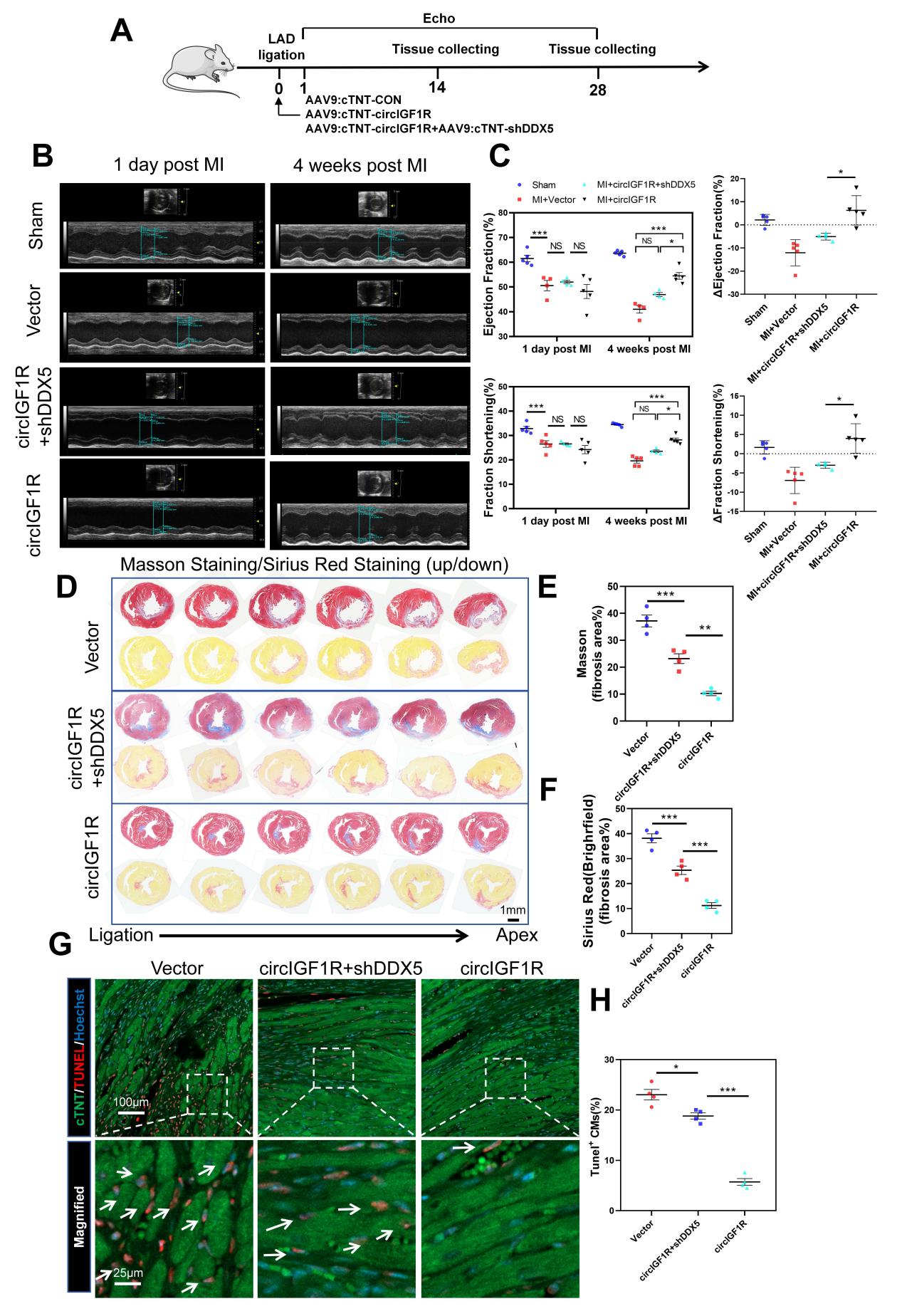
**

**Figure S9. CircIGF1R overexpressing promotes adult cardiac regeneration and improves myocardium repair after MI through DDX5.**

1. Experimental pattern: AAV9: cTNT-CON or AAV9: cTNT-circIGF1R or AAV9: cTNT-circIGF1R+AAV9: cTNT-shDDX5 was injected into myocardium following MI in P56 mice. Hearts were harvested at 14 and 28 dpi to evaluate cardiomyocyte proliferation, apoptosis and scar area, respectively. Echocardiography was performed at 1 and 28 dpi to detect cardiac function. (B and C) Cardiac function of ejection fraction and fractional shortening among the sham, AAV9: cTNT-CON, AAV9: cTNT-circIGF1R and AAV9: cTNT-circIGF1R+AAV9: cTNT-shDDX5 treated mice at 1 and 28 day post operation were detected by echocardiography, n=4 or 5 in each group. (D-F) Masson’s trichrome staining and Sirius red staining were used to determine scar formation among the AAV9: cTNT-CON, AAV9: cTNT-circIGF1R and AAV9: cTNT-circIGF1R+AAV9: cTNT-shDDX5 treated mice at 28 dpi, n=4 in each group. Scale bars, 1mm. (G and H) Representative pictures and quantification analysis of CM apoptosis quantified by TUNEL staining in infarct border zone in AAV9: cTNT-CON, AAV9: cTNT-circIGF1R and AAV9: cTNT-circIGF1R+AAV9: cTNT-shDDX5 groups after MI, n=4 in each group. Scale bars, 100 μm. Scale bars, 25 μm. Picture in each rectangular box was enlarged in the neighboring right panel. Data are presented as mean ± SEM. ns: no significance. *P ≤0.05. **P≤0.01. ***P ≤0.001

**Supplementary Table S1:** The primers used in this study.

| Primers name |  | Sequence (5’-3’) |
| --- | --- | --- |
| circIGF1R (human) | F | GCTGCCAGAAAATCTGCGG |
|  | R | ATGTGGAGGTAGCCCTCGAT |
| DDX5 (human) | F | ATGTCGGGTTATTCGAGTGACC |
|  | R | TGTGCGCCTAGCCAAATCAG |
| GAPDH (human) | F | GGAGTATGGTTGCAAAGCTGA |
|  | R | ATCTGTCAATCCTGTCCGTGT |
| circIGF1R (mouse)  Divergent Primers 1 | F | CTGTTACCTCTCCACCATAG |
|  | R | GGGCCCACAGATTTTCTGGCAG |
| circIGF1R (mouse)  Divergent Primers 2 | F | CTGCCAGAAAATCTGTGGGCCC |
|  | R | AAGCGGTAGCTTCGGTAGTC |
| circIGF1R (mouse)  Convergent Primers | F | TCCACATCCTGCTCATCT |
|  | R | TCGGCGTTCTTCTCAATC |
| Linear-IGF1R mRNA (mouse) | F | GTGGGGGCTCGTGTTTCTC |
|  | R | GATCACCGTGCAGTTTTCCA |
| DDX5 (mouse) | F | TCTCGGGATGACACCA |
|  | R | GTCCACTCGGGCTTTA |
| GAPDH (mouse) | F | CGGGATGACACCACTC |
|  | R | GTCCACTCGGGCTTTA |
| U6 (mouse) | F | GCGCGTCGTGAAGCGTTC |
|  | R | GTGCAG GGTCCGAGGT |

**Supplementary Table S2:** Identified circIGF1R binding proteins through by mass spectrometry following circRNA pull-down.

| prot_acc | gene name | score | mass | cover | pi | emPAI |
| --- | --- | --- | --- | --- | --- | --- |
| sp\|P50446\|K2C6A_MOUSE | Krt6a | 195 | 59641 | 9.8 | 8.04 | 0.24 |
| tr\|Q3SYP5\|Q3SYP5_MOUSE | Krt16 | 114 | 51973 | 9.8 | 5.13 | 0.2 |
| tr\|A0A2R8VHF3\|A0A2R8VHF3_MOUSE | Gm49450 | 95 | 30027 | 16.1 | 5.23 | 0.37 |
| tr\|B2RRX1\|B2RRX1_MOUSE | Actb | 94 | 42052 | 9.9 | 5.29 | 0.25 |
| tr\|Q546G4\|Q546G4_MOUSE | Alb | 84 | 70700 | 2.1 | 5.75 | 0.05 |
| tr\|A0A1B0GSG5\|A0A1B0GSG5_MOUSE | Rnh1 | 74 | 55659 | 4.1 | 4.92 | 0.06 |
| tr\|G3X922\|G3X922_MOUSE | Dnajc13 | 63 | 256559 | 2.2 | 6.32 | 0.01 |
| tr\|S4R1W5\|S4R1W5_MOUSE | Rbm6 | 48 | 128981 | 4.3 | 5.8 | 0.05 |
| sp\|Q62470\|ITA3_MOUSE | Itga3 | 47 | 117869 | 2.5 | 6.13 | 0.03 |
| tr\|H3BIZ7\|H3BIZ7_MOUSE | Frmd4a | 44 | 115949 | 1.6 | 8.98 | 0.03 |
| tr\|A1L333\|A1L333_MOUSE | Ddx5 | 41 | 69750 | 3.7 | 9.11 | 0.1 |
| tr\|A0A075B5P4\|A0A075B5P4_MOUSE | Ighg1 | 39 | 36292 | 8.3 | 6.88 | 0.09 |
| tr\|Q0VG47\|Q0VG47_MOUSE | Hnrnpa3 | 39 | 37291 | 9 | 8.46 | 0.19 |
| tr\|A0A0A6YW67\|A0A0A6YW67_MOUSE | Gm8797 | 38 | 8723 | 11.7 | 6.56 | 0.4 |
| tr\|A0A0R4J0Q4\|A0A0R4J0Q4_MOUSE | Loxl1 | 38 | 67139 | 6.8 | 6.94 | 0.1 |
| tr\|A0A1Y7VKY1\|A0A1Y7VKY1_MOUSE | Rps18-ps5 | 37 | 17738 | 7.2 | 10.85 | 0.19 |
| tr\|A0A1L1SRC3\|A0A1L1SRC3_MOUSE | Rbm7 | 37 | 17288 | 27.8 | 9.43 | 0.43 |
| tr\|A2A7K5\|A2A7K5_MOUSE | Mycbp | 37 | 16718 | 6.2 | 9.11 | 0.2 |
| tr\|A0A087WSM1\|A0A087WSM1_MOUSE | Ica1l | 37 | 49775 | 1.8 | 5.09 | 0.07 |
| sp\|P09055\|ITB1_MOUSE | Itgb1 | 37 | 91424 | 1.5 | 5.68 | 0.04 |
| tr\|F6Q3E1\|F6Q3E1_MOUSE | Prrc2b | 32 | 24812 | 6.5 | 5.39 | 0.13 |
| tr\|D3Z4N4\|D3Z4N4_MOUSE | Igf2 | 32 | 11556 | 8.7 | 5.36 | 0.3 |
| tr\|A0A1D5RM17\|A0A1D5RM17_MOUSE | Ndrg4 | 31 | 11445 | 14.7 | 6.33 | 0.3 |
| tr\|A0A2I3BQF4\|A0A2I3BQF4_MOUSE | Rpl30 | 31 | 10621 | 29.8 | 9.32 | 0.32 |
| sp\|P32020\|SCP2_MOUSE | Scp2 | 30 | 59715 | 1.8 | 7.16 | 0.06 |
| tr\|D3Z6F5\|D3Z6F5_MOUSE | Atp5f1a | 30 | 54675 | 2.8 | 8.24 | 0.06 |
| sp\|Q9D832\|DNJB4_MOUSE | Dnajb4 | 29 | 37929 | 4.7 | 8.7 | 0.09 |
| sp\|Q5GH68\|XKR2_MOUSE | Xkrx | 28 | 52315 | 2.7 | 8.88 | 0.06 |
| tr\|A0A0U1RPW2\|A0A0U1RPW2_MOUSE | Tjp1 | 28 | 127400 | 1.7 | 6.23 | 0.03 |
| tr\|E9PU96\|E9PU96_MOUSE | Urb1 | 26 | 256791 | 0.7 | 6.5 | 0.01 |
| tr\|F6R7E8\|F6R7E8_MOUSE | Gm2663 | 25 | 27243 | 8.1 | 6.49 | 0.12 |
| sp\|Q6P1E8\|EFCB6_MOUSE | Efcab6 | 24 | 177086 | 1.3 | 9.02 | 0.02 |
| tr\|E9QMD3\|E9QMD3_MOUSE | Zfhx3 | 24 | 410697 | 0.5 | 5.84 | 0.01 |
| tr\|G3X9W0\|G3X9W0_MOUSE | Hnrnpd | 23 | 36356 | 4.2 | 8.14 | 0.09 |
| sp\|Q3UH06\|RREB1_MOUSE | Rreb1 | 21 | 187003 | 0.6 | 6.28 | 0.02 |
| sp\|Q9R118\|HTRA1_MOUSE | Htra1 | 18 | 52094 | 2.3 | 7.82 | 0.06 |

**Supplementary Table S3:** Information about antibodies used in this article.

| Antibody | Dilution | Application | Catalogue no. | Company |
| --- | --- | --- | --- | --- |
| cTnT | 1:200 | IF | ab8295 | Abcam |
| Ki67 | 1:200 | IF | ab16667 | Abcam |
| Aurora B | 1:200 | IF | ab2254 | Abcam |
| pH3 | 1:200 | IF | 9701 | CST |
| Hoechst33342 | 1:200 | IF | H3570 | Thermo Fisher |
| Alexa Fluor 488 | 1:200 | IF | A11001 | Invitrogen |
| Alexa Fluor 647 | 1:200 | IF | A11008 | Invitrogen |
| WGA | 1:200 | IF | w32466 | Thermo Fisher |
| DDX5 | 1:800 | WB | ab126730 | Abcam |
| CCND1 | 1:800 | WB | 26939-1-AP | Proteintech |
| c-myc | 1:800 | WB | 10828-1-AP | Proteintech |
| β-catenin | 1:800 | WB | 51067-2-AP | Proteintech |
| GAPDH | 1:800 | WB | ab8245 | Abcam |
