# Supplementary figures and images for: Circular RNA IGF1R Promotes Cardiac Repair via Activating β-Catenin Signaling by Interacting with DDX5 in Mice after Ischemic Insults
Source: Research (Wash D C). 2024 Aug 27;7:0451. doi: 10.34133/research.0451 (PMC11347128; doi:10.34133/research.0451)

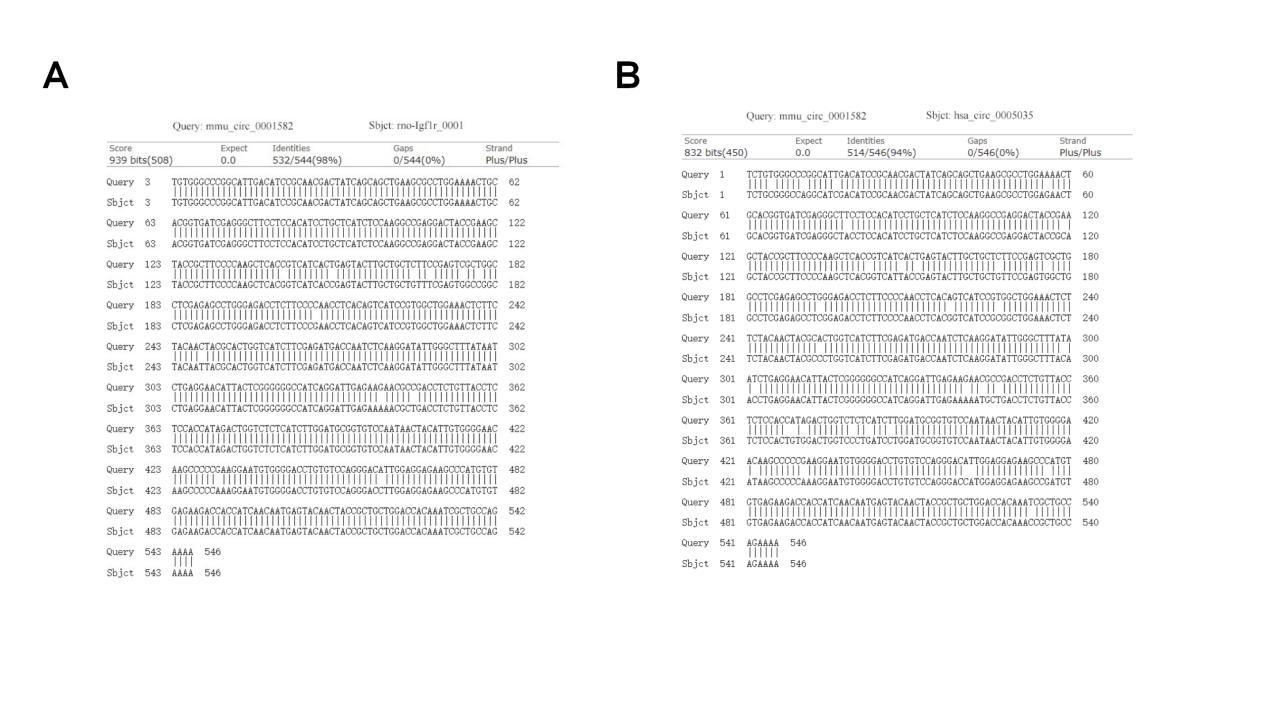

Supplement: Supplementary 1 — Materials and Methods Figs. S1 to S9 Tables S1 to S5 [file research.0451.f1.zip › Figure S1.jpg]

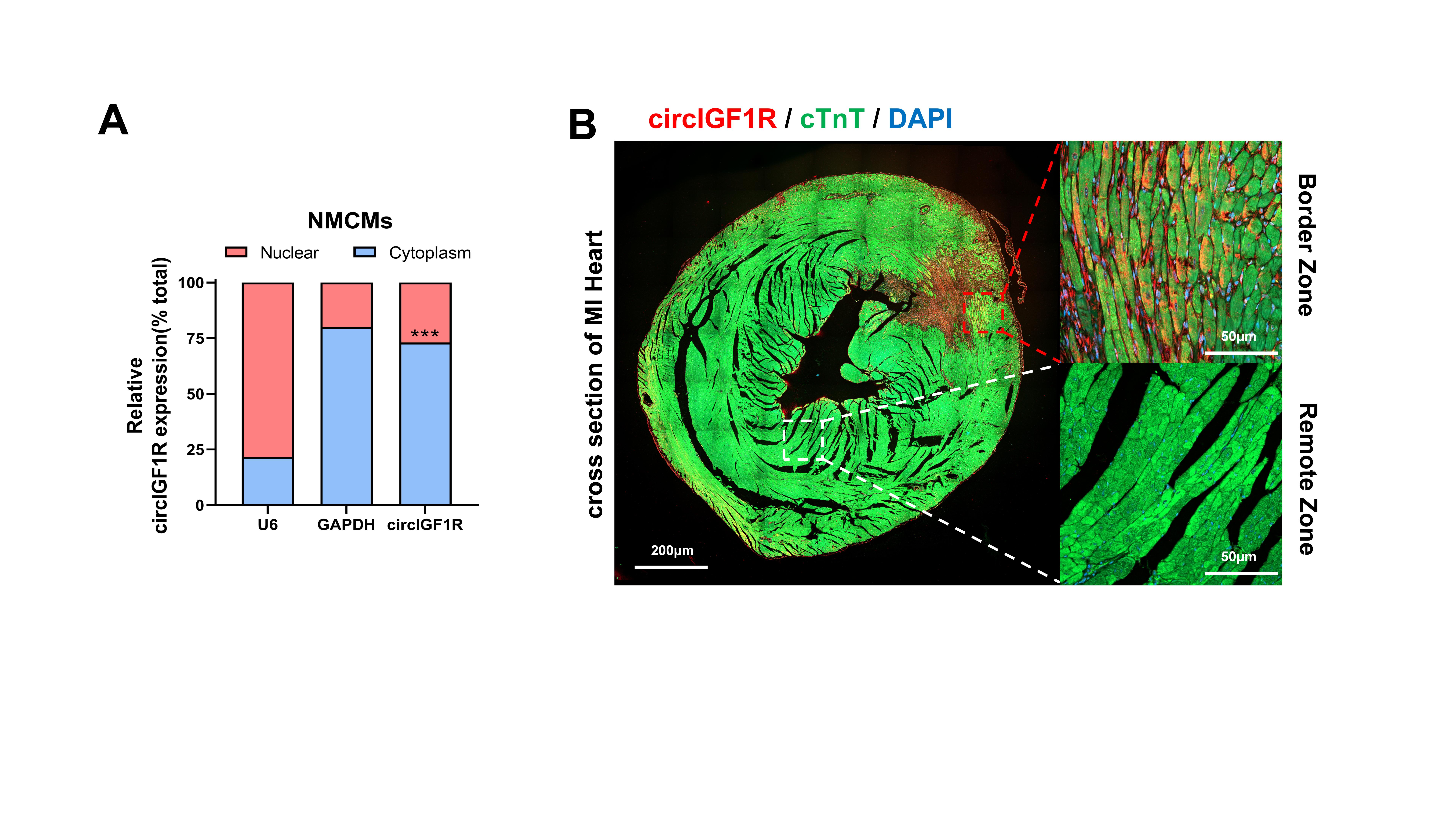

Supplement: Supplementary 1 — Materials and Methods Figs. S1 to S9 Tables S1 to S5 [file research.0451.f1.zip › Figure S2.jpg]

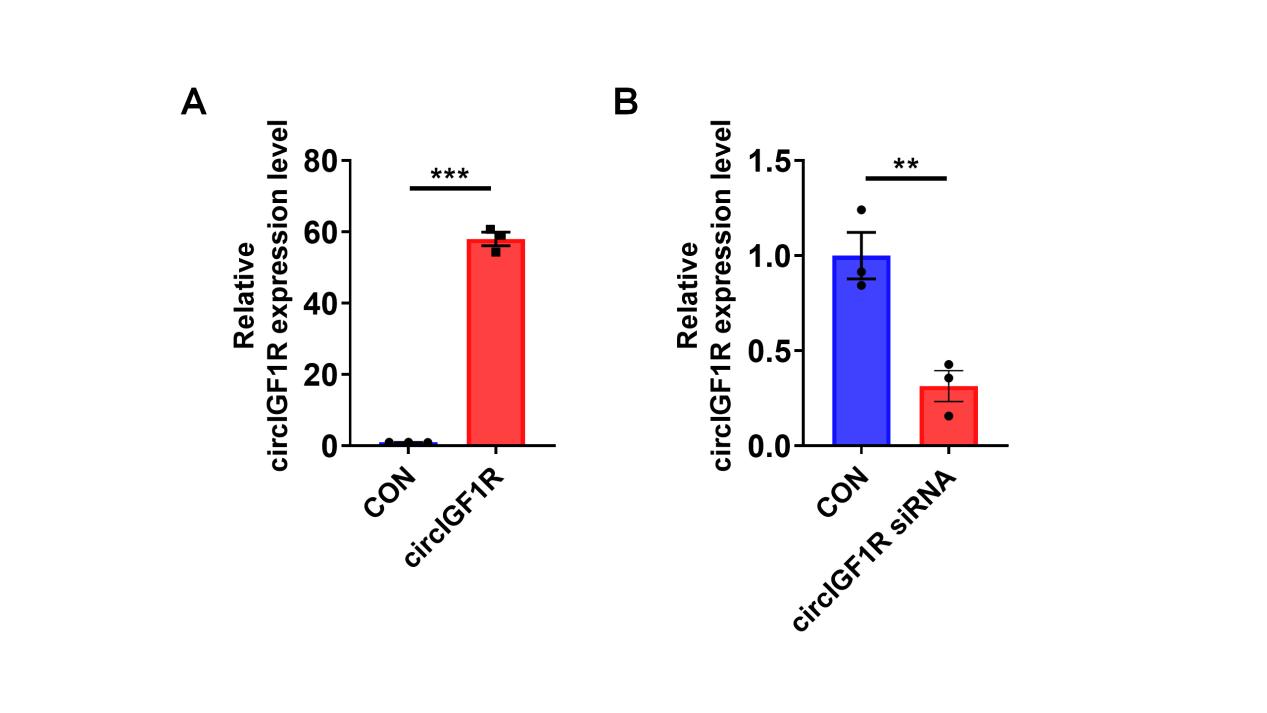

Supplement: Supplementary 1 — Materials and Methods Figs. S1 to S9 Tables S1 to S5 [file research.0451.f1.zip › Figure S3.jpg]

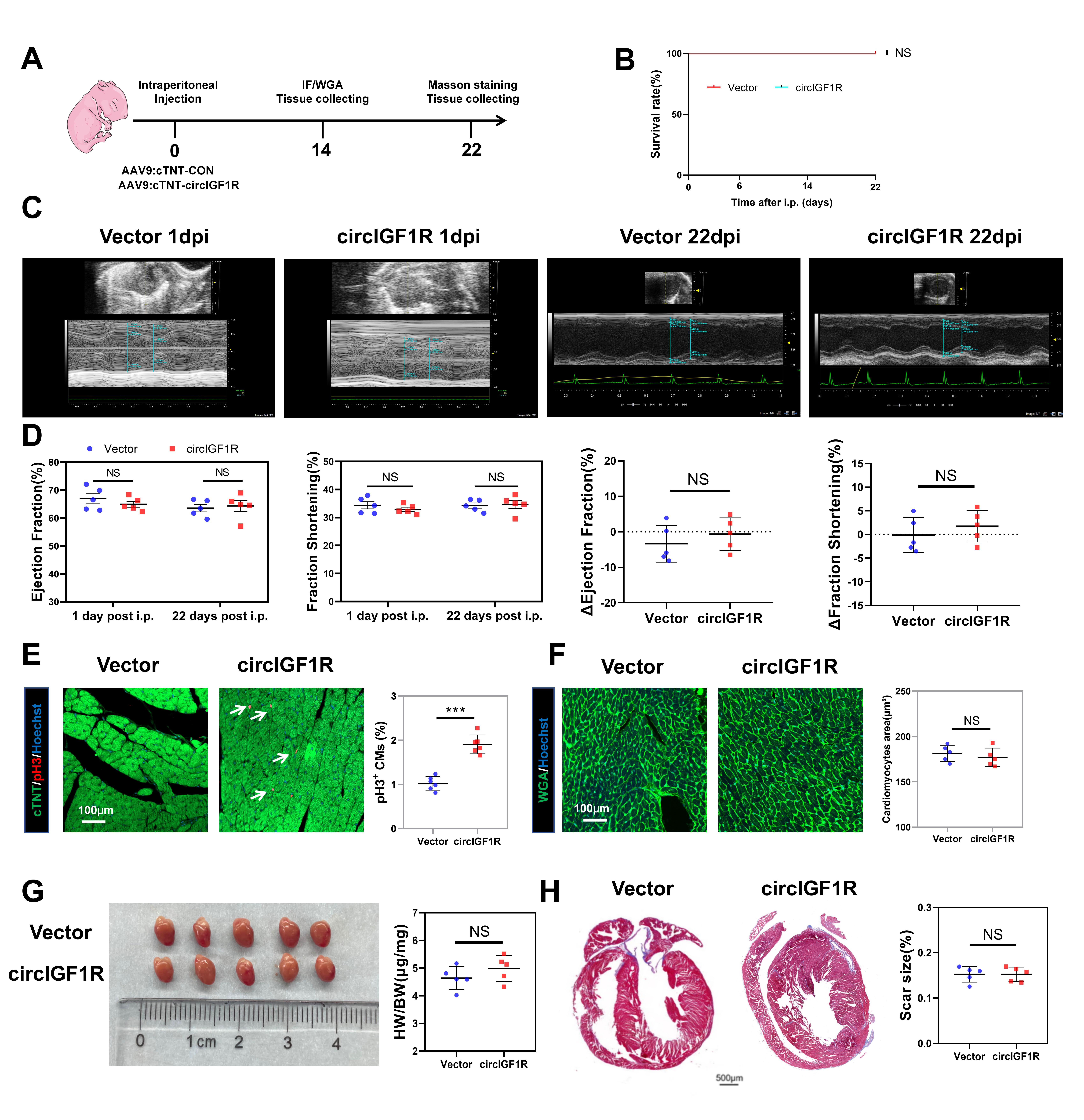

Supplement: Supplementary 1 — Materials and Methods Figs. S1 to S9 Tables S1 to S5 [file research.0451.f1.zip › Figure S4.jpg]

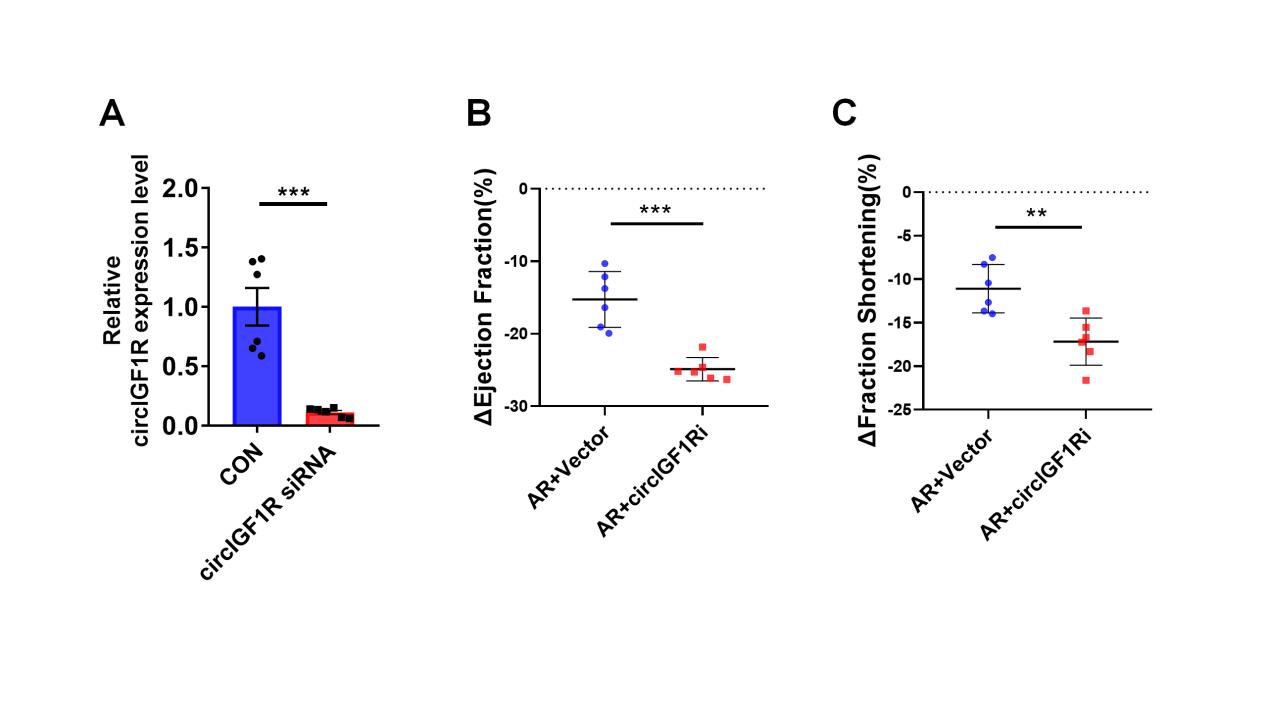

Supplement: Supplementary 1 — Materials and Methods Figs. S1 to S9 Tables S1 to S5 [file research.0451.f1.zip › Figure S5.jpg]

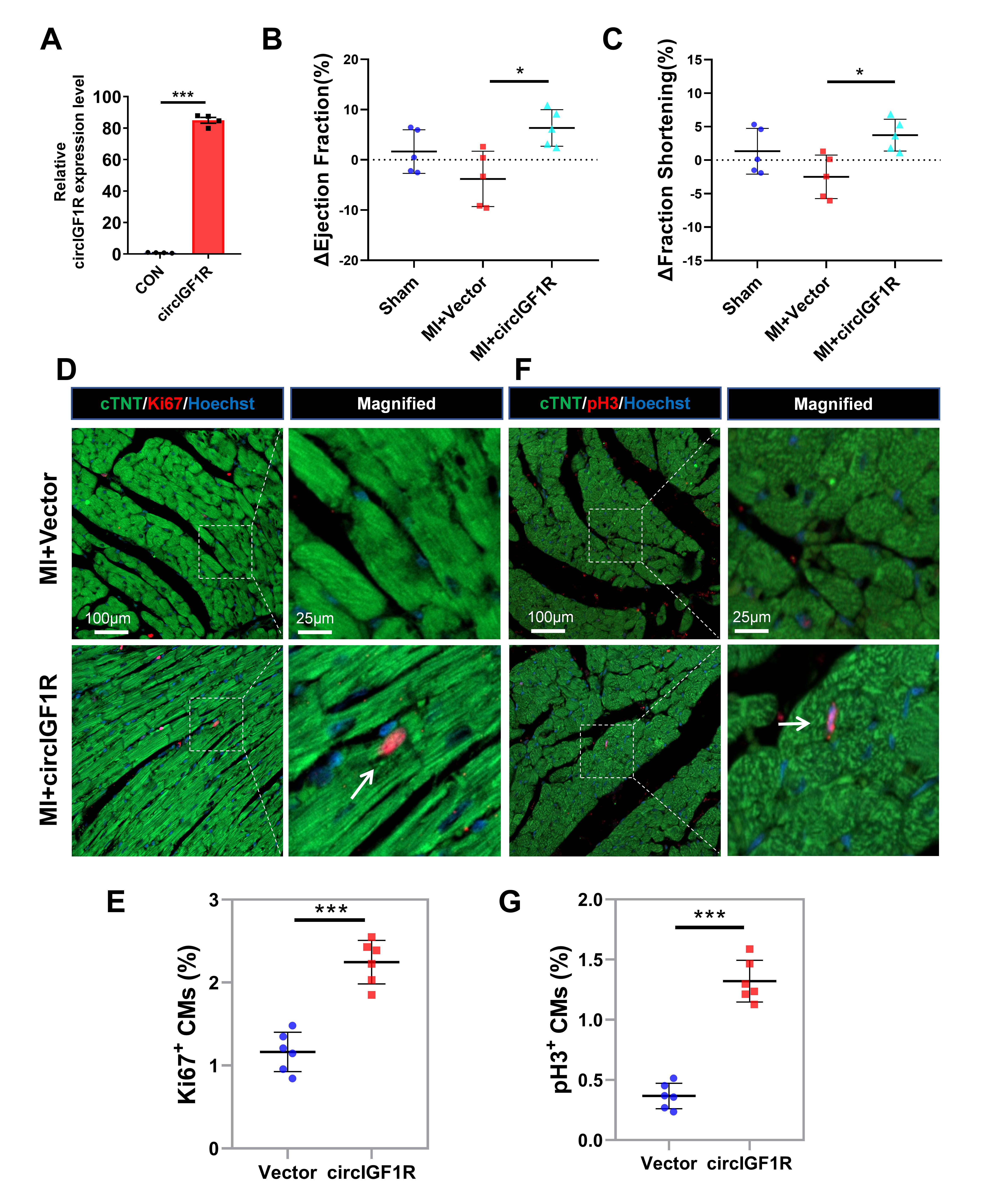

Supplement: Supplementary 1 — Materials and Methods Figs. S1 to S9 Tables S1 to S5 [file research.0451.f1.zip › Figure S6.jpg]

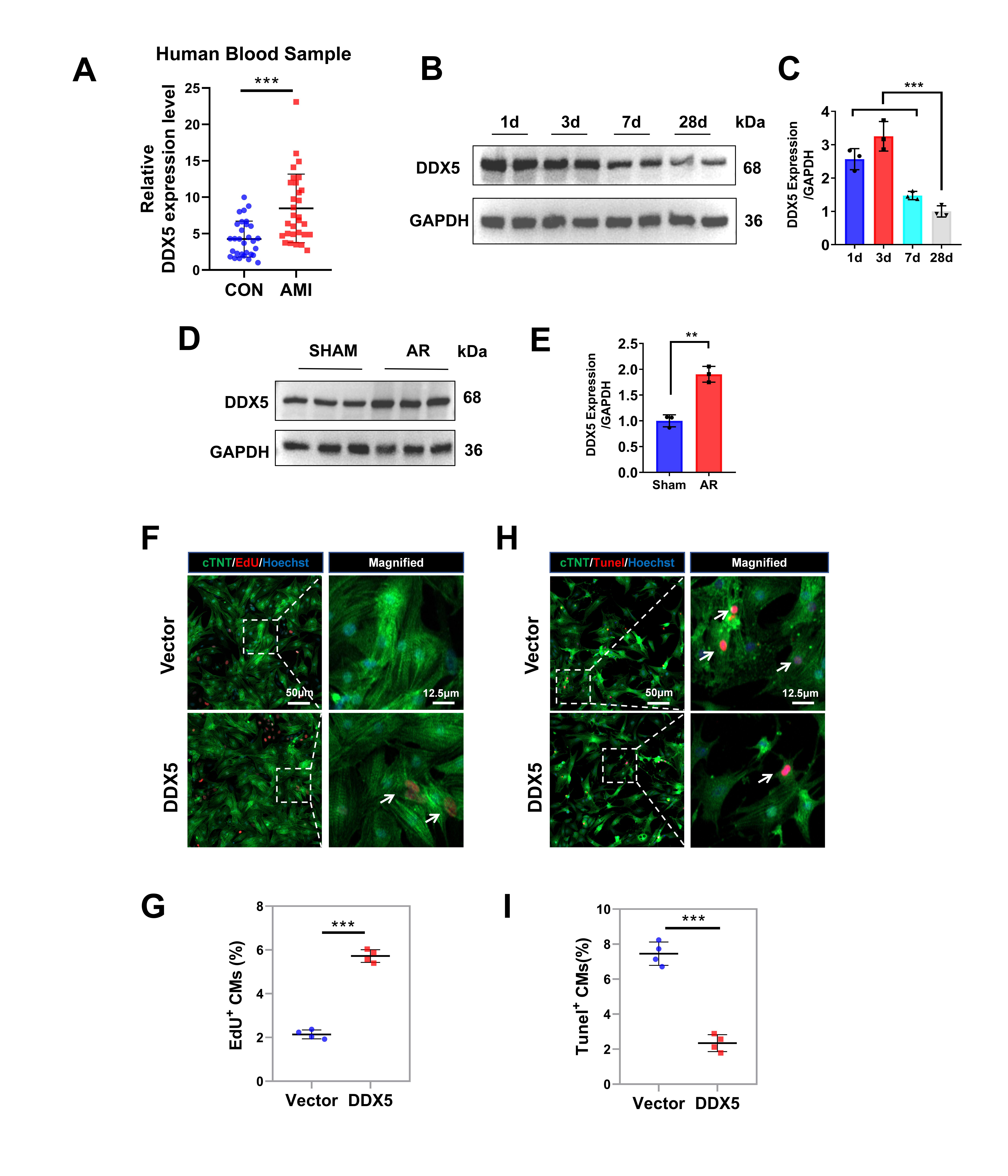

Supplement: Supplementary 1 — Materials and Methods Figs. S1 to S9 Tables S1 to S5 [file research.0451.f1.zip › Figure S7.jpg]

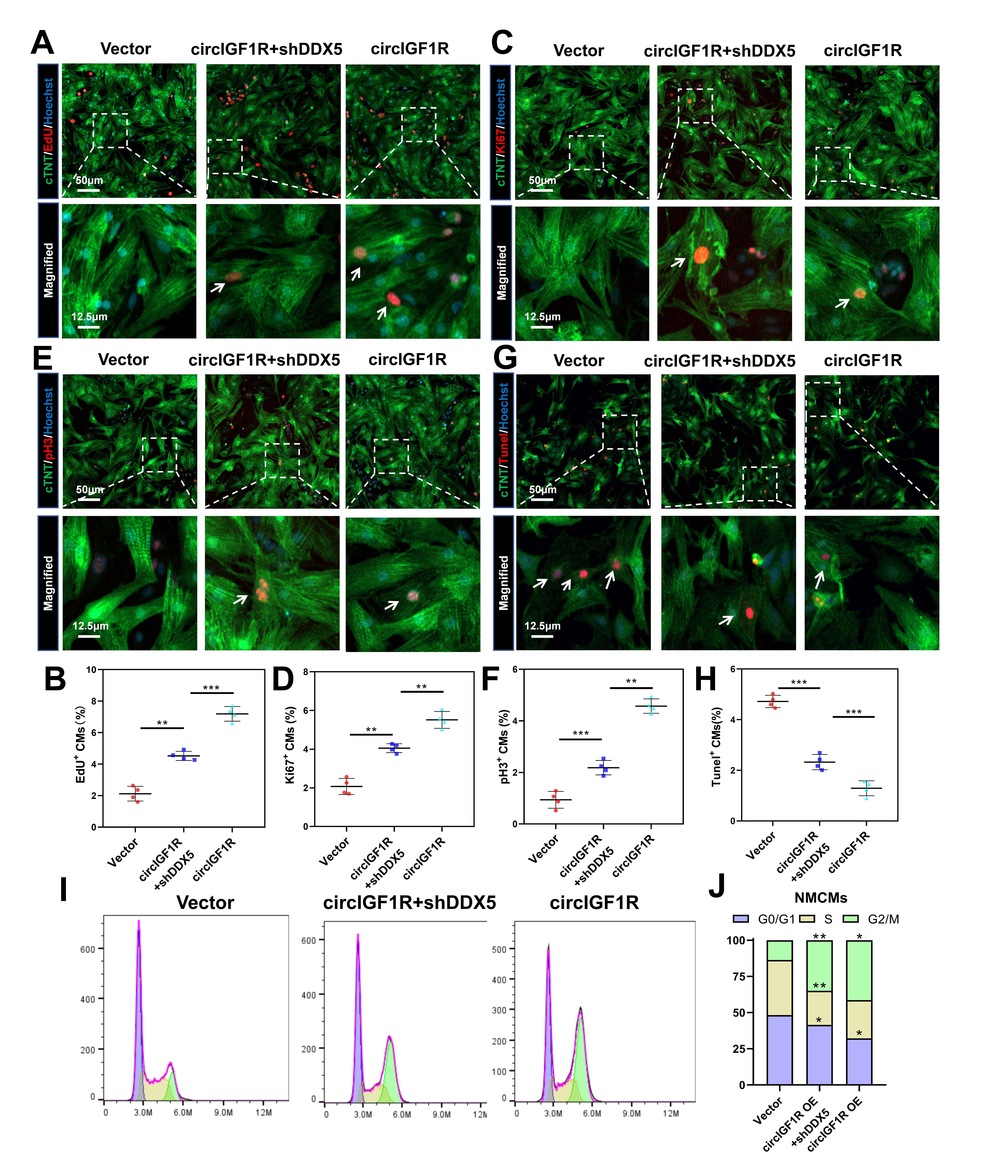

Supplement: Supplementary 1 — Materials and Methods Figs. S1 to S9 Tables S1 to S5 [file research.0451.f1.zip › Figure S8.jpg]

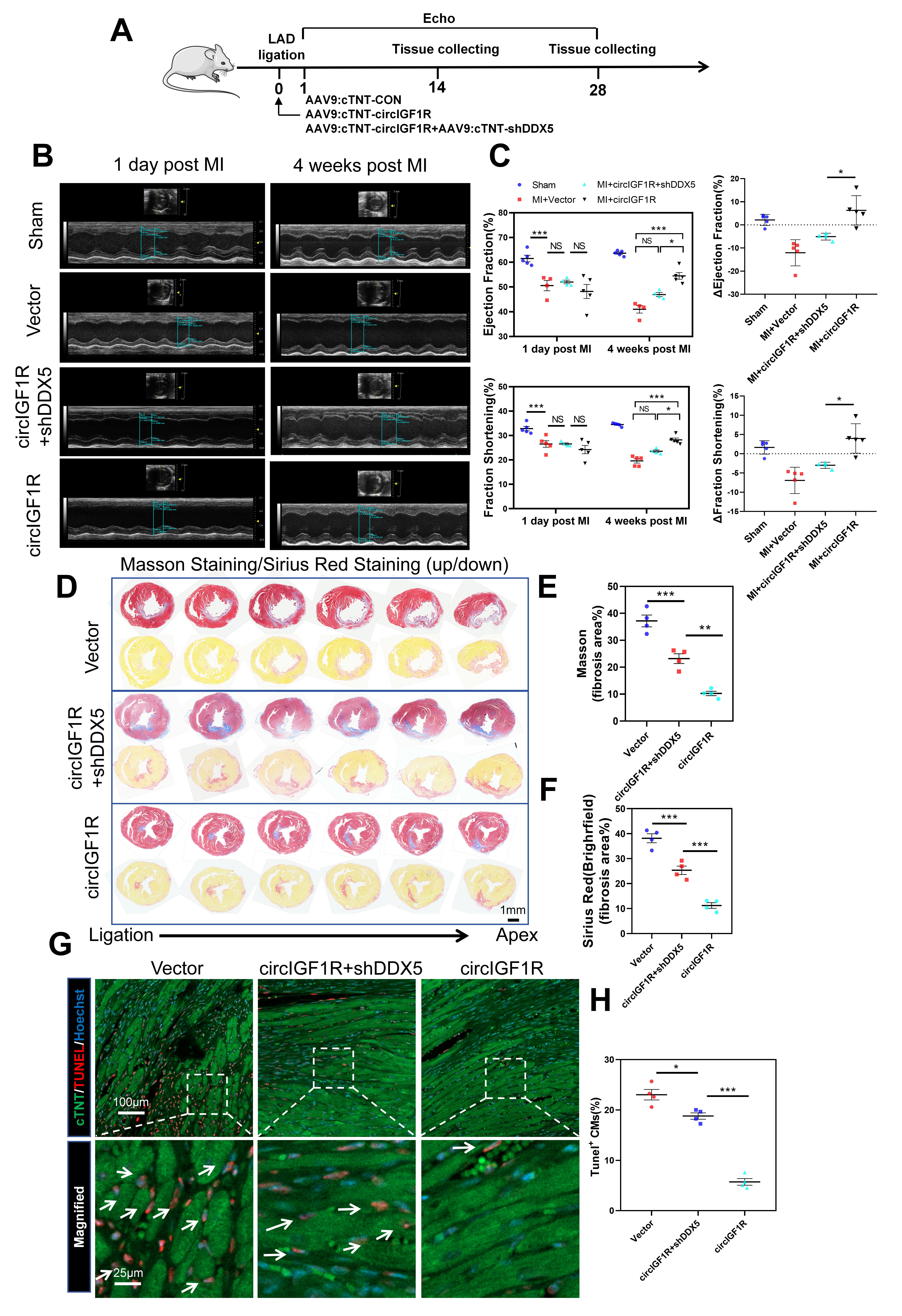

Supplement: Supplementary 1 — Materials and Methods Figs. S1 to S9 Tables S1 to S5 [file research.0451.f1.zip › Figure S9.jpg]
